# Links Between Altered Feedback Learning and Symptoms of Depression: Insights From the FRN and Feedback‐Locked N170

**DOI:** 10.1111/psyp.70228

**Published:** 2026-01-09

**Authors:** Madita Röhlinger, Julian Vahedi, Christian Bellebaum

**Affiliations:** ^1^ Faculty of Mathematics and Natural Sciences, Institute for Experimental Psychology Heinrich Heine University Düsseldorf Düsseldorf Germany

**Keywords:** delayed feedback, depression, FRN/RewP, N170, prediction error

## Abstract

Blunted electrophysiological and striatal responses to reward have been suggested as biomarkers or endophenotypes for depression. However, previous studies did not differentiate between learning from immediate and learning from delayed feedback, which involves different neural structures. The aim of the present study was to clarify whether depression alters learning from both immediate and delayed feedback. We investigated the influence of current and past depressive symptom severity and familial history of depression in a mixed clinical and nonclinical sample of 45 individuals on two event‐related potential (ERP) components, namely, the feedback‐related negativity (FRN) and N170. In the past, the FRN has been reported to primarily reflect the processing of immediate feedback, while the N170—most commonly studied in relation to face processing—appeared to represent the processing of delayed feedback. We found that performance in a probabilistic feedback learning task with immediate and delayed feedback was reduced for more severe depressive symptoms, regardless of feedback timing. Surprisingly, the FRN was not affected by current or past depressive symptom severity or familial vulnerability to depression. However, we found depression‐related changes in the N170 for both immediate and delayed feedback processing: currently experienced depressive symptoms were associated with poorer encoding of prediction errors in the N170. In addition, a family history of depression was associated with lower sensitivity to the valence of feedback in the N170. In summary, the N170 may emerge as a novel, important biomarker in clinical research on depression and feedback‐based learning.

## Impact Statement

Previous studies reported negative effects of depression on learning from feedback. In the present study, we could not replicate findings of blunted feedback‐related negativity (FRN) amplitudes in depression but found poorer encoding of prediction errors in the N170 related to depression. In addition, we found a blunted N170 feedback valence sensitivity linked to a familial history of depression. Thus, the N170 might serve as a biomarker in research on depression and feedback‐based learning.

While modern living conditions seem to feed the incidence of depression, many questions concerning its pathophysiology are still unresolved (Hidaka 2012). Researchers try to find structural and functional alterations in the brain that may help explain the underlying mechanisms of depression. Given the heterogeneity of the symptoms, it is likely that multiple brain regions and mechanisms are involved (Nestler et al. 2002; Thompson 2023). The etiological diversity of depression is so complex that it can hardly be studied in its entirety (Kendler et al. 2002). Therefore, recent research has focused on identifying endophenotypes, hoping to better understand biological mechanisms contributing to depression. Endophenotypes are inheritable traits that allow linking observable symptoms with genetic predispositions and thereby help to develop tailored interventions (Luking et al. 2016).

In this context, it was suggested that dysfunctional reward processing is a crucial aspect in the pathophysiology of depression (Admon and Pizzagalli 2015) and that blunted neural responses to reward might be an endophenotype for depression (Bress et al. 2015; Luking et al. 2016). The dopaminergic midbrain forms the core of the brain's reward system (Björklund and Dunnett 2007; Glimcher 2011; Haber and Knutson 2010; Schultz and Dickinson 2000). However, midbrain dopaminergic neurons do not signal reward itself; rather, they reflect whether an outcome is better or worse than expected, encoding a prediction error (PE; Schultz et al. 1997). PE signals have also been found to be reflected in brain activity measured via electroencephalography (EEG). More precisely, the feedback‐related negativity (FRN) is an event‐related potential (ERP) component that peaks between 230 and 330 ms after feedback onset (Miltner et al. 1997). While its amplitude was reported to be larger for losses than gains, more recent research suggests that the negative going waveform is the baseline response and that amplitude modulations of the component rather reflect a positivity following rewards, leading to the conceptualization of the reward positivity (RewP; Holroyd et al. 2008; for a review, see Proudfit 2015). This is supported by studies reporting that the signal in the time window of the FRN/RewP especially reflects a positive PE (Kirsch et al. 2022; Weber and Bellebaum 2024). However, there are also studies that indicate that the signal in the FRN/RewP time window is modulated by negative PEs (Röhlinger, Albrecht, and Bellebaum 2025; Röhlinger, Albrecht, Ghio, et al. 2025). Therefore, we refer to the component as FRN, well aware that it also reflects a relative positivity following rewards. Given its modulation by PEs, this electric brain signal measured over the scalp might reflect influences of the mesencephalic dopaminergic reward system on the anterior cingulate cortex, which is a likely generator of the FRN (Bellebaum and Daum 2008; Foti et al. 2011; Holroyd et al. 2004; Holroyd and Coles 2002; Oerlemans et al. 2025). However, many brain areas encode PEs with different functional roles (Den Ouden et al. 2012) and there is no direct evidence showing that the FRN is affected by activity of midbrain dopaminergic neurons (Jocham and Ullsperger 2009; Ullsperger et al. 2014).

Numerous studies describe a link between depression and reduced feedback valence sensitivity in the FRN, driven by reduced (less positive) amplitudes following rewards (Bress et al. 2012, 2013, 2015; Foti et al. 2014; Klawohn et al. 2021; for a meta‐analytic review see Keren et al. 2018), even in young preschool‐age children (Belden et al. 2016). In line with changes in the FRN, altered and mainly impaired feedback learning has been reported for depressed individuals (Admon et al. 2017; Bakic et al. 2017; Kumar et al. 2018; Kunisato et al. 2012; Macoveanu et al. 2014; Pechtel et al. 2013; Pizzagalli et al. 2005, 2008; for a review see Chen et al. 2015 and Eshel and Roiser 2010). However, previous studies did not differentiate between learning from immediate and learning from delayed feedback, the processing of which involves different neuronal structures. The processing of immediate feedback is based on the striatum (Foerde et al. 2013; Foerde and Shohamy 2011), and striatal hypo‐functioning is decisive for dysfunctional reward processing in depression (Pizzagalli et al. 2009; Takamura et al. 2017; for a review see Admon and Pizzagalli 2015 and Luking et al. 2016). Within nondepressed and depressed individuals, the ERPs in the FRN time window and striatal activation were correlated, indicating convergence across the two measures (Becker et al. 2014; Carlson et al. 2011; Foti et al. 2014).

In contrast, the processing of delayed feedback has been suggested to rely less on the striatum (Foerde et al. 2013; Foerde and Shohamy 2011). Several ERP studies (Arbel et al. 2017; Höltje and Mecklinger 2020; Peterburs et al. 2016; Weinberg et al. 2012; Weismüller and Bellebaum 2016) support this by describing a decrease in the amplitude difference between positive and negative feedback for the FRN following delayed feedback. At the same time, an increase in the amplitude of the N170 ERP component has been described with increased feedback delay (Arbel et al. 2017; Höltje and Mecklinger 2020; Kim and Arbel 2019). While the N170 is most often studied in relation to the perceptual processing of visual stimuli, particularly faces (Bentin et al. 1996; for a review see Yovel 2016), this component also plays a role in a broader set of functions, such as spatial navigation and encoding reward locations (Baker and Holroyd 2009, 2013; Baker et al. 2015). Considering its pronounced amplitudes in response to delayed feedback, the feedback‐locked N170 may be related to stronger hippocampal processing for delayed feedback in the context of reinforcement learning (Foerde et al. 2013, Foerde and Shohamy 2011). As this ERP component is most pronounced over the occipitotemporal cortex in a time window between 100 and 200 ms, it was interpreted as reflecting processes in the medial temporal lobe (MTL), which includes the hippocampus (Arbel et al. 2017; Höltje and Mecklinger 2020; Kim and Arbel 2019; Raslau et al. 2015). A recent study suggested that regions within the MTL initiate the reactivation of representations of visual stimuli in higher order visual areas to link them to delayed feedback and that this mechanism of credit assignment is reflected in the feedback‐locked N170 (Röhlinger, Albrecht, and Bellebaum 2025). In the same study, it was found that the feedback‐locked N170 reflects the whole range of PEs, with more pronounced amplitudes for unexpected than expected positive feedback and smaller amplitudes following unexpected than expected negative feedback (Röhlinger, Albrecht, and Bellebaum 2025). This has been interpreted as reflecting the need to reactivate or recall the events that led to an unexpected reward in order to guide future behavior.

Interestingly, the hippocampus—which may be a potential generator of the feedback‐locked N170—also plays a role in etiological models linking depression to chronic stress: Stress, whether acute or chronic, activates the hypothalamic–pituitary–adrenal axis, and extreme or long‐lasting stress can cause damage to the hippocampus (for a review, see Nestler et al. 2002). Accordingly, depression can be accompanied by hippocampal atrophy, which in turn is linked to memory impairment and might contribute to some of the cognitive distortions seen in depression (Fairhall et al. 2010; Nestler et al. 2002; Thompson 2023). Besides weaknesses in recollection, depressed individuals tend to have a reduced memory for positive events, while their memory for negative events is increased (Shah et al. 1998; for a review see Dillon and Pizzagalli 2018). Findings by Hager et al. (2021) suggest that depression is associated with dysfunctional source memory for rewards but not losses. Given the broad symptoms of depression, it is no surprise that the pathophysiology involves a variety of brain regions, including the hippocampus and striatum (Nestler et al. 2002).

Although the processing of immediate and delayed feedback seems to primarily rely on different structures in the brain, it is conceivable that also for delayed feedback depression is accompanied by behavioral and neurophysiological changes. Based on the changes in the hippocampus and related functions, we expected that depressed individuals would also show alterations in learning from and processing of delayed feedback, with the latter being reflected in different neurophysiological signals, i.e., the N170, compared to immediate feedback. With the present work, we aimed to investigate the link between depression and the behavioral performance as well as the electrophysiological processing in a probabilistic feedback learning task with immediate and delayed feedback.

It must be taken into account that endophenotypes are state‐independent, i.e., they can be detected in a person even if the disease is not active (Gottesman and Gould 2003). Accordingly, blunted striatal activity was found in remitted depressed individuals (McCabe et al. 2009) as well as blunted (more negative) FRN amplitudes following rewards in “healthy” siblings of depressed individuals (Weinberg et al. 2015). There is also an increased risk of developing depression for the children of depressed mothers (Halligan et al. 2007; Raposa et al. 2014), accompanied by blunted responses to reward within the dorsal and ventral striatum, relative to children of nondepressed mothers (for an extensive review see Luking et al. 2016). In summary, depressed individuals, remitted individuals, and those at high risk show blunted striatal responses to the receipt of (immediate) rewards. Therefore, we assessed current depressive symptoms, past depression episodes, and the family history of depression as predictors for feedback learning and processing. Since dichotomizing a continuous variable leads to a loss of information and reduced statistical power (see Clayson et al. 2020), we approach depression not as a binary state (healthy vs. depressed) but as a dimensional construct, operationalized as a continuous variable (see also Hager et al. 2021) in a mixed clinical and nonclinical sample.

Because there is evidence for a publication bias and only a weak relationship between FRN and depression has been found in previous studies (Clayson et al. 2020; Moran et al. 2017), the first aim of the planned study is to replicate findings of reduced learning performance and FRN amplitude with immediate feedback in the context of depression. In several studies, the association between depression and reduced feedback valence sensitivity in the FRN was mainly driven by the response to reward (Belden et al. 2016; Bress et al. 2012, 2013, 2015; Brush et al. 2018; Foti et al. 2014). Therefore, we expected a reduced sensitivity to feedback valence in the FRN in individuals with an increased familial vulnerability for depression, individuals that have experienced depressive episodes in the past and participants that currently experience depressive symptoms, especially in the immediate feedback condition. In addition, we aimed to investigate whether these individuals also show alterations like reduced amplitudes for the N170, especially following delayed feedback. Finally, for both ERP components, we aimed to explore depression‐related changes in the neural processing of the PE, possibly in interaction with feedback timing.

## 1 Method

### 1.1 Participants

The sample size was based on the number of participants in previous studies. Bress et al. (2015) found a significant, moderate to strong correlation (*r* = 0.41, *p* < 0.010) between depressive symptomatology and the valence sensitivity in the FRN time window (i.e., smaller amplitudes of the negative–positive feedback difference wave) in a sample of 41 individuals. Taking dropouts due to EEG artifacts or exclusion criteria (see below) into account, we preregistered to recruit 50 participants (18 to 40 years). Exclusion criteria were current or former neurological disorders, acute psychotic conditions, the regular or acute consumption of substances affecting the central nervous system, knowledge about Hiragana‐Characters, and uncorrected impaired vision. To consider acute depressive symptoms as a continuous factor in the analysis and therefore cover sufficient variance, we promoted the study on the campus of the Heinrich Heine University Düsseldorf, via social media platforms and at the Outpatient Psychotherapy Unit of the LVR Clinic for Psychosomatic Medicine and Psychotherapy in Düsseldorf. In total, we acquired data from 50 participants. We excluded five participants, three of them because they fulfilled at least one of our exclusion criteria, one because of bad EEG data quality determined during visual inspection of the raw data, and one due to improper setup of the EEG system during the acquisition. The final sample included in the analyses thus consisted of 45 participants: 37 reported being women and eight men; 41 were right‐handed, three left‐handed, and one ambidextrous. The mean age was 24.87 years (SD = 5.54 years, Min = 18 years, Max = 39 years).

### 1.2 Procedure

Upon arrival in the laboratory, participants were informed about the experimental procedure and gave written informed consent to participate in the study, followed by a short clinical interview (see below) lasting about 30 min. Afterwards, participants were placed in front of a 27 in, 1920 × 1080 px W‐LED monitor (BENQ EW2740L) with a refresh rate of 60 Hz and filled in a demographic questionnaire that additionally contained questions about past depressive episodes (see below) and whether a first‐degree relative is or was affected by depression (see below), followed by a questionnaire to assess the participants' current depressive symptoms (see below). Then we attached EEG electrodes and started the experimental task (see below) which lasted about 45 min. Participation was compensated with 5€ per 30 min or course credit for psychology students. Additionally, participants received the money they earned during the feedback learning task (see below) rounded up to 6€. The study was approved by the ethics committee of the Faculty of Mathematics and Natural Sciences at Heinrich Heine University Düsseldorf and in accordance with the declaration of Helsinki.

### 1.3 Preceding Interview

To detect psychological disorders apart from or comorbid with depression, a trained experimenter conducted parts of the Mini‐DIPS (Margraf and Cwik 2017; Margraf et al. 2017) with all participants (for a similar approach see Bress et al. 2013; Foti et al. 2014). The Mini‐DIPS is an abbreviated version of the Diagnostic Interview for Psychological Disorders (DIPS) and, according to Margraf and Cwik (2017), offers an efficient and reliable diagnosis of psychological disorders according to DSM‐5 and ICD‐10 for research questions. The interview begins with a set of questions designed to screen for symptoms, and if any are affirmed, additional questions are asked to evaluate if the symptoms fulfill the necessary criteria for a diagnosis. We used the Mini‐DIPS to detect the following conditions: anxiety disorders, substance addiction disorders, eating disorders, obsessive‐compulsive disorder, affective disorders, and suicidality. We decided to screen for these disorders because they pose common comorbidities in depression (Jacobi et al. 2014; Lamers et al. 2011; Zimmerman et al. 2002), were in some cases associated with the FRN (Aarts and Pourtois 2012; Bellato et al. 2021; Forester et al. 2024; Gu et al. 2010; Jiang et al. 2018; Ryu et al. 2017; Sehrig et al. 2019; Takács et al. 2015; Tobias and Ito 2021) and are some of the most common psychological disorders in adulthood in Germany (Suhr 2020).

### 1.4 Assessment of Depression

#### 1.4.1 Familial Vulnerability

We asked participants whether a first‐degree relative, i.e., parent or sibling (excluding half‐siblings), has ever been diagnosed with depression, providing “yes,” “no,” and “I'm not sure” as response options.

#### 1.4.2 Past Depressive Episodes

Adapted from the approach by Bress et al. (2013), we used a modified version of the mood module of the Patient Health Questionnaire (PHQ‐9; Kroenke et al. 2001; German version: Gräfe et al. 2004) to evaluate past depressive episodes. The modified version by Cannon et al. (2007) contains 9 items (e.g., *Little interest or pleasure in doing things*) that can be rated on a 4‐point scale ((0) *not at all* to (3) *almost every day*). Importantly, the modified version refers to the 2 weeks in the participants' lives when they were feeling most blue, sad, or depressed. The sum score can range from 0 to 27, with higher values indicating more severe depressive symptoms. High correspondence with lifetime diagnosis based on the Structured Clinical Interview for DSM–IV (SCID; First and Gibbon 2004) makes the modified PHQ‐9 an efficient measure of lifetime depression (Cannon et al. 2007).

#### 1.4.3 Current Depressive Symptoms

We used the Beck Depression Inventory (BDI‐II; Beck et al. 1996; German version: Hautzinger et al. 2006) as a measure for acute depression severity (for a similar account, see Bress et al. 2013). It contains 21 items, each consisting of 4 statements reflecting values from 0 to 3. For example, the first item addresses sadness with the following statements: (0) *I do not feel sad* (1) *I feel sad much of the time* (2) *I am sad all of the time* (3) *I am so sad or unhappy that I can't stand it*. We asked participants to indicate which of the four statements has most likely been true for them in the past 2 weeks. The values of the ticked statements were summed up and built a score ranging from 0 (no depressive symptomatology) to 63 (severe symptomatology).

### 1.5 Experimental Task and Conditions

**FIGURE 1:**
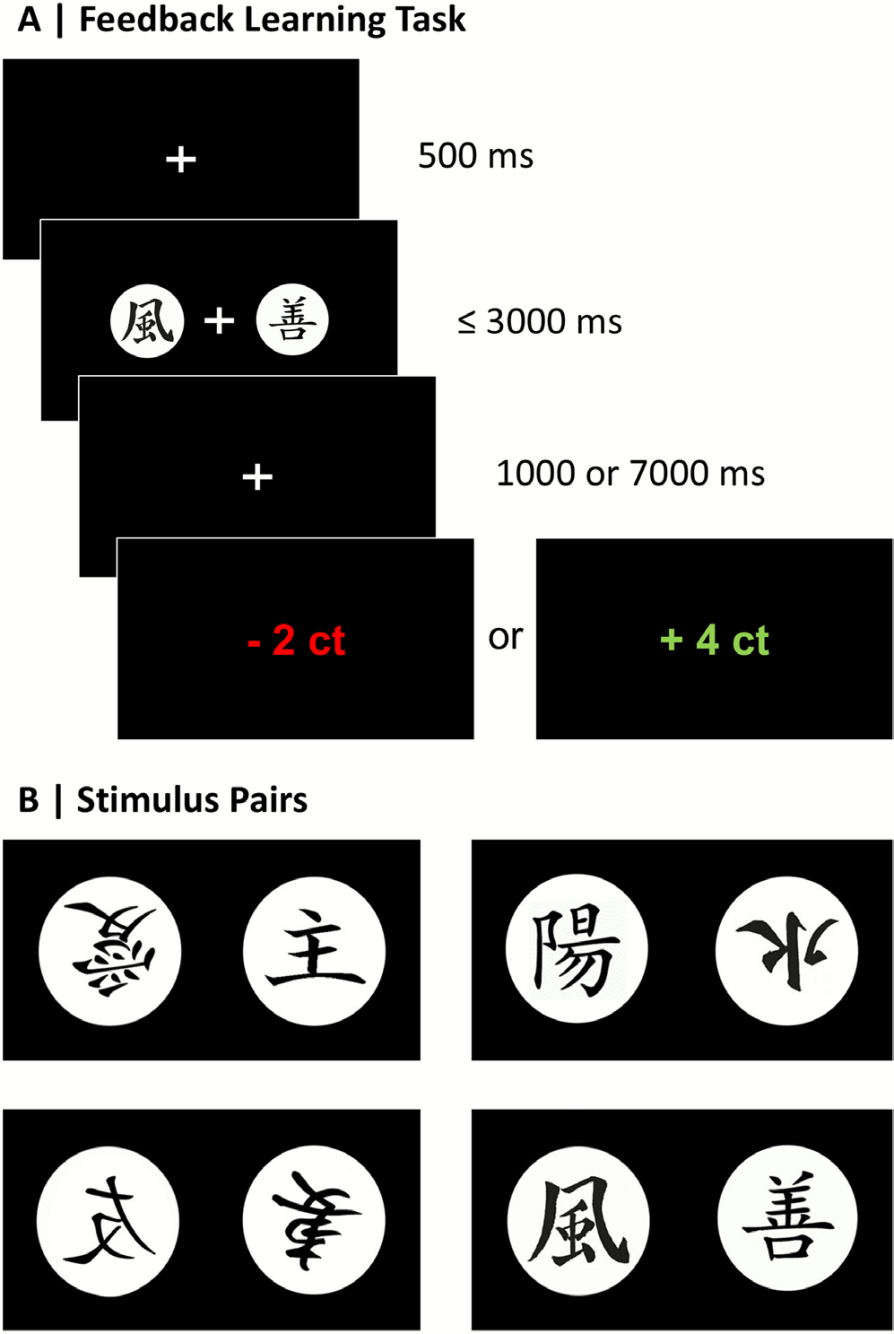
Stimuli and time course of the probabilistic feedback learning task. (A) Feedback Learning Task: The assignment of visual stimuli to the left and right side of the screen, as well as the side on which the more rewarding stimulus was presented, was counterbalanced to ensure that feedback could clearly be associated with a stimulus and not with a response side. (B) Stimulus Pairs: The neighboring stimuli build the four pairs used for all participants. The stimulus associated with higher reward probability was randomly determined when a new pair was presented.

Participants performed a probabilistic feedback learning task, where they could learn associations between stimuli and positive (+4ct) or negative (−2ct) monetary feedback (see Figure 1A). The task contained the within‐subject factor feedback timing: Feedback appeared either 1 s (immediate feedback) or 7 s (delayed feedback) after the participant's choice. The experiment consisted of four learning phases, two with immediate and two with delayed feedback. Learning phases with immediate versus delayed feedback alternated, and it was counterbalanced to determine which feedback timing condition was presented first.

Figure 1A shows the temporal sequence of a trial, from the presentation of two available stimuli to the selection and feedback. The software Presentation (Neurobehavioral Systems Inc., Albany, CA, USA) controlled the timing of stimulation and the recording of responses. Responses were performed on a standard computer keyboard (Logitech K120) where participants could press the left and right control keys to choose between the stimuli. One stimulus of each pair was associated with reward in 65% of the trials and with punishment in 35%, while the probabilities were reversed for the other stimulus. The participant's task was to learn which stimulus was more likely to be rewarded and thus maximize the reward. Participants were instructed that wins and losses contribute to the total amount of money paid out at the end. A new pair of stimuli was presented in each of the four learning phases (see Figure 1B). Each learning phase consisted of 4 blocks of 20 trials with short breaks in between, i.e., 80 trials per learning phase and 320 trials in total.

### 1.6 EEG Data Acquisition

We acquired EEG data from a total of 60 electrodes, fixed with an actiCap textile softcap (BrainProducts, Germany), and evenly distributed on the scalp based on the extended 10–20 system. Electrodes were attached to the scalp sites AF3, AF4, AF7, AF8, C1, C2, C3, C4, C5, C6, CP1, CP2, CP3, CP4, CP5, CP6, CPz, Cz, F1, F2, F3, F4, F5, F6, F7, F8, FC1, FC2, FC3, FC4, FC5, FC6, FT10, FT7, FT8, FT9, Fz, O1, O2, Oz, P1, P2, P3, P4, P5, P6, P7, P8, PO10, PO3, PO4, PO7, PO8, PO9, POz, Pz, T7, T8, TP7, TP8. In addition, the ground electrode was attached to the AFz position and an online reference to the position FCz. We placed two further electrodes over the left and right mastoids to cover as much of the scalp as possible for the average reference (see below). Finally, we attached two more electrodes (vEOG) above (at Fp1 position) and below the left eye to keep track of vertical eye movements and blinks. A BrainAmp DC amplifier (BrainProducts, Germany) and the Brain Vision Recorder software (BrainProducts, Germany) were used for data recording with a sampling rate of 1000 Hz and an online lowpass filter of 100 Hz. We kept impedances below 15 kΩ.

### 1.7 Data Analysis

#### 1.7.1 Behavioral Analysis

We performed generalized linear mixed‐effects models (GLME) analyses suitable for binomial distributions and single‐trial data using the lme4 package (version 1.1.34; Bates et al. 2015) in R (The R Foundation 2021). The dependent variable was response accuracy, with correct responses (defined as the choice of the stimulus associated with the higher reward probability) coded as 1 and incorrect responses as 0. We calculated three separate models, one for each measure of depression as a predictor. The first model comprised the following fixed‐effect predictors: the BDI (between‐subjects: severity of current depressive symptoms measured via the BDI‐II), Feedback Timing (within‐subjects: immediate vs. delayed), and Block (1–4; because learning is indicated by an increase in the number of correct responses within the same learning phase), together with all interactions. Participants were included as random intercepts. For the inclusion of random‐effect slopes per participant, we considered best practice guidelines (Meteyard and Davies 2020): we included all within‐subject main and interaction effects as random slopes, unless their inclusion compromised model fit. The maximal model was determined by using the buildmer (Version 2.11; Voeten 2020) function.

The other two models were constructed based on the same principle, with the only difference being the depression measure used as fixed‐effect predictor. The second model contained the PHQ (a measure of past depressive episodes assessed through the modified PHQ‐9) and the third model included Familial Vulnerability for depression as a predictor (binary categorical variable indicating whether a first‐degree relative has ever been diagnosed with depression). The resulting model formulas for all three models are presented in Table S1 of the Supporting Information.

#### 1.7.2 Modeling of Prediction Errors

We inferred single‐trial values of the PE for each participant by fitting a reinforcement learning model to the behavioral data in MATLAB (version R2021a, The MathWorks Inc. 2021; for a similar approach see Burnside et al. 2019; Lefebvre et al. 2017; Röhlinger, Albrecht, and Bellebaum 2025; Röhlinger, Albrecht, Ghio, et al. 2025; Weber and Bellebaum 2024). The basis for the application of the reinforcement learning model was each participants' sequence of choices and the feedback they received. The PE $\delta_{c,t}$ was conceptualized as:

$$\delta_{c,t}=r_{t}-Q_{c,t}$$

where in a given trial t the reward $r_{t}$ is 1 for positive feedback and 0 for negative feedback, and $Q_{c,t}$ is the value of the stimulus the participant chose. Separately for each of the four stimulus pairs, we primarily assigned both stimuli a stimulus value of 0.5, that was iteratively adjusted in every trial t in which the stimulus pair was displayed. The stimulus value of the chosen stimulus, $Q_{c}$, was adjusted based on the difference between the previous value and the obtained outcome (the PE δ), together with a learning rate α that mirrors how much the participant used the PE to adjust the stimulus value. For each of the four stimulus pairs, we modeled different learning rates for learning from positive feedback and negative feedback, as this procedure consistently yielded better fitting models than a procedure with only one learning rate for both positive and negative feedback (see Röhlinger, Albrecht, and Bellebaum 2025; Röhlinger, Albrecht, Ghio, et al. 2025). We adjusted the stimulus value of the chosen stimulus with the learning rate $\alpha_{\mathrm{con}}$ for trials with positive feedback that confirms the choice as follows:

$$Q_{c,t+1}=Q_{c,t}+\alpha_{\mathrm{con}}\times \delta_{c,t}$$

For trials with negative feedback that disconfirms the choice, the stimulus value of the chosen stimulus was adjusted with the learning rate $\alpha_{\mathrm{dis}}$:

$$Q_{c,t+1}=Q_{c,t}+\alpha_{\mathrm{dis}}\times \delta_{c,t}$$

As both stimuli of a pair were always displayed together, we expected that participants would form assumptions about the unchosen stimulus from feedback for the chosen stimulus. Therefore, the value of the unchosen stimulus, $Q_{u},$ was 1‐$Q_{c}$ and was updated accordingly, as in our previous studies, in a better model fit was reached with this procedure compared to a procedure without updating of the value of the unchosen stimulus (see Röhlinger, Albrecht, and Bellebaum 2025; Röhlinger, Albrecht, Ghio, et al. 2025).

For each trial, $t_{1,\ldots ,n_{\text{trials}}}$, we calculated the probability p that the model would choose the stimulus which was indeed chosen by the participant with the help of the softmax function. This calculation was based on prior stimulus values of both stimuli that were displayed, namely values of the chosen stimulus, $Q_{c,t}$, and the unchosen stimulus in trial t, $Q_{u,t}$, along with an exploration parameter β:

$$p_{c,t}=\frac{e^{\mathrm{Qc},t\times \beta }}{e^{\mathrm{Qc},t\times \beta }+e^{\mathrm{Qu},t\times \beta }}$$

with β indicating how much prior stimulus values affected the participants choices. A larger β indicates that a participant relied more on earlier stimulus values, whereas a smaller β indicates that the participant was more explorative in the choice behavior.

In a next step, we used the probabilities p to calculate the negative summed log‐likelihood $\left(-\mathrm{LL}\right)$ as measure for the model's goodness of fit:

$$-\Sigma \;\log\left(p_{c,t_{1},\ldots ,n_{\text{trials}}}\right)$$

The optimization function fmincon from the Optimization Toolbox of MATLAB (R2021a, The MathWorks Inc. 2021) minimized the −LL value by estimating values for the free parameters ($\alpha_{\mathrm{con}},\alpha_{\mathrm{dis}},\beta$) that led to the least difference between the model's predicted choices and the participant's actual behavior. The model was fit repeatedly (50 iterations) to the participants' behavior to prevent convergence to local minima. We allowed random numbers within the interval [0; 1] as start values for the free parameters. We set boundaries of [0; 1] for the learning rates ($\alpha_{\mathrm{con}},\alpha_{\mathrm{dis}}$), and [0; 100] for the exploration parameter (β).

To examine how well the chosen model captured the actual choice behavior, we performed a posterior predictive check, which is illustrated in Figure S1 in the Supporting Information. Descriptively, the empirical data and the behavior predicted by the model are consistent in capturing learning, though note that the model marginally underpredicted the actual task performance, particularly in later blocks. The mean −LL for the best fitting model across participants was 158.30 (SD = 85.31, Min = 27.40, Max = 428.77). The model revealed a mean learning rate of 0.36 (SD = 0.25, Min = 0.0004, Max = 1.00) for $\alpha_{\mathrm{con}}$ and 0.15 (SD = 0.21, Min = 0.001, Max = 0.97) for $\alpha_{\mathrm{dis}}$. For a more detailed overview of model fit, learning rates and exploration parameters per participant see Figure S2 in the Supporting Information. In Addition, Table S2 in the Supporting Information shows results from a correlational analysis between the latent variables ($\alpha_{\mathrm{con}},\alpha_{\mathrm{dis}},\beta$), derived from the computational PE modeling and the depression variables (BDI, PHQ, Familial Vulnerability). However, learning rates and exploration parameters were not related to depression.

#### 1.7.3 EEG Data Analysis

BrainVision Analyzer 2.2 (Brain Products GmbH 2018), MATLAB R2021a (The MathWorks Inc. 2021), and R (The R Foundation 2021) were used for EEG data analysis. Trials in which participants failed to answer (*M* = 1.00%, SD = 1.60%, Min = 0.00%, Max = 8.44%) were excluded from any further EEG analyses.

##### 1.7.3.1 Preprocessing

We first re‐referenced the data to the average of all scalp electrodes and calculated the signal at the online reference site FCz (for similar procedures see Arbel et al. 2017; Höltje and Mecklinger 2020; Röhlinger, Albrecht, and Bellebaum 2025; Röhlinger, Albrecht, Ghio, et al. 2025). To minimize the reduction of ERP effects that can result from using an average reference (see Luck 2014), we used high‐density EEG acquisition, including data from 63 scalp electrodes (see above, including mastoids) into the average reference. Afterward, we filtered the data with a 30 Hz low pass and a 0.1 Hz high pass filter (as proposed by Luck 2014) as well as a 50 Hz notch filter. Then, we performed an independent component analysis (ICA), followed by a reversed ICA on single‐subject EEG data to remove blinking artifacts. In the next step, we created segments from 200 ms before to 800 ms after feedback onset, followed by a baseline correction relative to the first 200 ms of the segment. Then, we excluded segments with artifacts in the electrodes of our interest (for similar approaches see Albrecht et al. 2023; Röhlinger, Albrecht, and Bellebaum 2025; Röhlinger, Albrecht, Ghio, et al. 2025), i.e., electrodes used to measure the FRN (Fz, FCz, Cz, FC1 & FC2) and N170 (P7 and P8). Precisely, all segments containing voltage steps > 50 μV/ms, differences between values > 80 μV or < 0.1 μV within an interval of 100 ms or amplitudes > 80 μV or < −80 μV were removed (*M* = 1.25%, SD = 3.25%, Min = 0.00%, Max = 17.41%). We grouped and averaged the remaining segments according to the conditions (positive and negative immediate feedback and delayed feedback), yielding four averages per participant. Eventually, we exported all single‐trial segment data as well as all averages per condition and participant for further analysis in MATLAB to extract single‐trial ERP data (MathWorks, MA).

For the FRN, we retrieved single‐trial amplitudes from the pooled signal of an electrode cluster consisting of Fz, FCz, Cz, FC1, and FC2 (for a similar approach see Röhlinger, Albrecht, and Bellebaum 2025; Röhlinger, Albrecht, Ghio, et al. 2025), as preregistered. First, we calculated the difference wave for negative—positive feedback separately for immediate and delayed feedback for each participant. We used the two difference waves to determine the maximum negative peak amplitude latency between 230 and 360 ms postfeedback, i.e., the time point where the signal differed the most between positive and negative feedback, for each participant. Then, for each single trial, we calculated the mean amplitude in a time window of ±10 ms around the condition‐specific peak latency. Importantly, the difference waves were only used to derive the latency of the time point when the difference between positive and negative feedback was maximal, but single‐trial values based on this latency were extracted from the original waveforms.

For the N170, we retrieved single‐trial amplitudes (see Albrecht et al. 2023) from electrodes P7 and P8 (for similar approaches see Arbel et al. 2017; Höltje and Mecklinger 2020; Kim and Arbel 2019; Röhlinger, Albrecht, and Bellebaum 2025; Röhlinger, Albrecht, Ghio, et al. 2025), as preregistered. First, we identified the maximum negative peak amplitude latency between 130 and 230 ms after feedback presentation in each participant's averages, at both electrode sites and for all four conditions separately (see above). Then, for each single trial, we calculated the mean amplitude in a time window of ±10 ms around the condition‐ and electrode‐specific N170 peak latency. In addition, we extracted the mean amplitude in a time window of ±10 ms around the preceding positive peak (P1). Similar to the approach used for the negative peak, P1 latency was determined using the condition‐specific average at each electrode site. We determined the P1 as the maximum positivity in a time window starting 75 ms after feedback onset to the respective condition‐specific negative peak. As dependent variable for the analysis, we used the N170 defined as the peak‐to‐peak amplitude by subtracting the single‐trial amplitude value corresponding to the preceding P1 from the single‐trial value corresponding to the negative peak (for a similar approach see Röhlinger, Albrecht, and Bellebaum 2025).

### 1.8 Statistical Analysis

#### 1.8.1 FRN

We analyzed the single‐trial FRN amplitude as the dependent variable in an LME analysis using R (Bates et al. 2015). Similar to the analysis of the behavioral data, we constructed three separate models, one for each of the three different depression variables as predictors (BDI, PHQ, and Familial Vulnerability). The first model contained the fixed‐effect predictors BDI (severity of current depressive symptoms measured via the BDI‐II), as well as Feedback Timing (immediate vs. delayed), Feedback Valence (negative vs. positive), the unsigned PE (indicating general expectation violations or surprise, independent of feedback valence), and all possible interactions between the factors. As a random‐effect factor, we included Participant. Random slopes per participant were added as described for the behavioral GLME above.

The other two models were created based on the same approach, with the only variation being the depression measure used as a fixed‐effect predictor. The second model contained the PHQ (a measure of past depressive episodes assessed through the modified PHQ‐9), and the third model included Familial Vulnerability for depression as a predictor (binary categorical variable indicating whether a first‐degree relative has ever been diagnosed with depression). The resulting model formulas for all three models are presented in Table S3 of the Supporting Information. To resolve significant interactions, simple slope analyses were performed with Bonferroni‐corrected *p*‐values (multiplied by the number of conducted tests).

#### 1.8.2 N170

We analyzed the single‐trial N170 amplitude as the dependent variable in an LME analysis using R (Bates et al. 2015). We followed the same approach as in the FRN analysis (see above), building three models that varied only regarding the depression variable used as a fixed‐effect predictor (BDI, PHQ, or Familial Vulnerability). As additional fixed‐effect predictors in each of the three models we added Feedback Timing (immediate vs. delayed), Feedback Valence (negative vs. positive), the unsigned PE (indicating general expectation violations or surprise, independent of feedback valence), Electrode (P7 vs. P8; because the N170 has been found to show hemispheric differences, see Röhlinger, Albrecht, and Bellebaum 2025), as well as all possible interactions between these factors. As random intercepts, we included Participant. Random slopes per participant were added as described for the behavioral GLME above. The formulas for the three resulting models are presented in Table S4 of the Supporting Information. Significant interactions were resolved as described for the FRN (see above).

## 2 Results

### 2.1 Mini‐Dips, BDI‐II, Modified PHQ‐9, and Familial Vulnerability

According to the Mini‐DIPS, depressive symptoms were the most frequent psychiatric symptoms in our sample. Of the 45 participants in our sample, 18 met the diagnostic criteria for a major depressive episode, either currently or in the past. 13 participants fulfilled the criteria for social anxiety disorder, and 11 participants met the criteria for generalized anxiety disorder, each either currently or in the past. Diagnostic criteria for other psychological disorders were met less frequently (see Table 1). Additionally, 9 participants reported having received outpatient or inpatient psychotherapy either currently or in the past. Participants reached a mean BDI score of 9.89 (SD = 9.34; Min = 0, Max = 39) and a mean PHQ score of 9.64 (SD = 5.68, Min = 0, Max = 26). Finally, 8 participants reported that a first‐degree relative has been diagnosed with depression, while this was not the case for 29 participants and another 8 participants were not sure.

**TABLE 1:** Descriptive statistics resulting from the Mini‐DIPS.

Diagnosis	*n*	Percent (%)
Anxiety disorders		
Panic disorder	4	8
Agoraphobia	4	8
Social anxiety disorder	13	26
Specific phobia	4	8
Gerneralized anxiety disorder	11	22
Affective disorders		
Manic episode	0	0
Hypomanic episode	6	12
Major depression	18	36
Persistent depressive disorder	3	6
Eating disorder		
Anorexia nervosa	3	6
Bulimia nervosa	5	10
Binge eating disorder	3	6
Obsessive‐compulsive disorder	4	8
Substance addiction	1	2
Suicidal tendencies	2	4

*Note:* *N* = 45. Mini‐DIPS = abbreviated version of the Diagnostic Interview for Psychological Disorders. Listed are the numbers of individuals who, according to their own reports during the interview, met the criteria for the psychological disorders presented, either currently or in the past.

### 2.2 Behavioral Results

**FIGURE 2:**
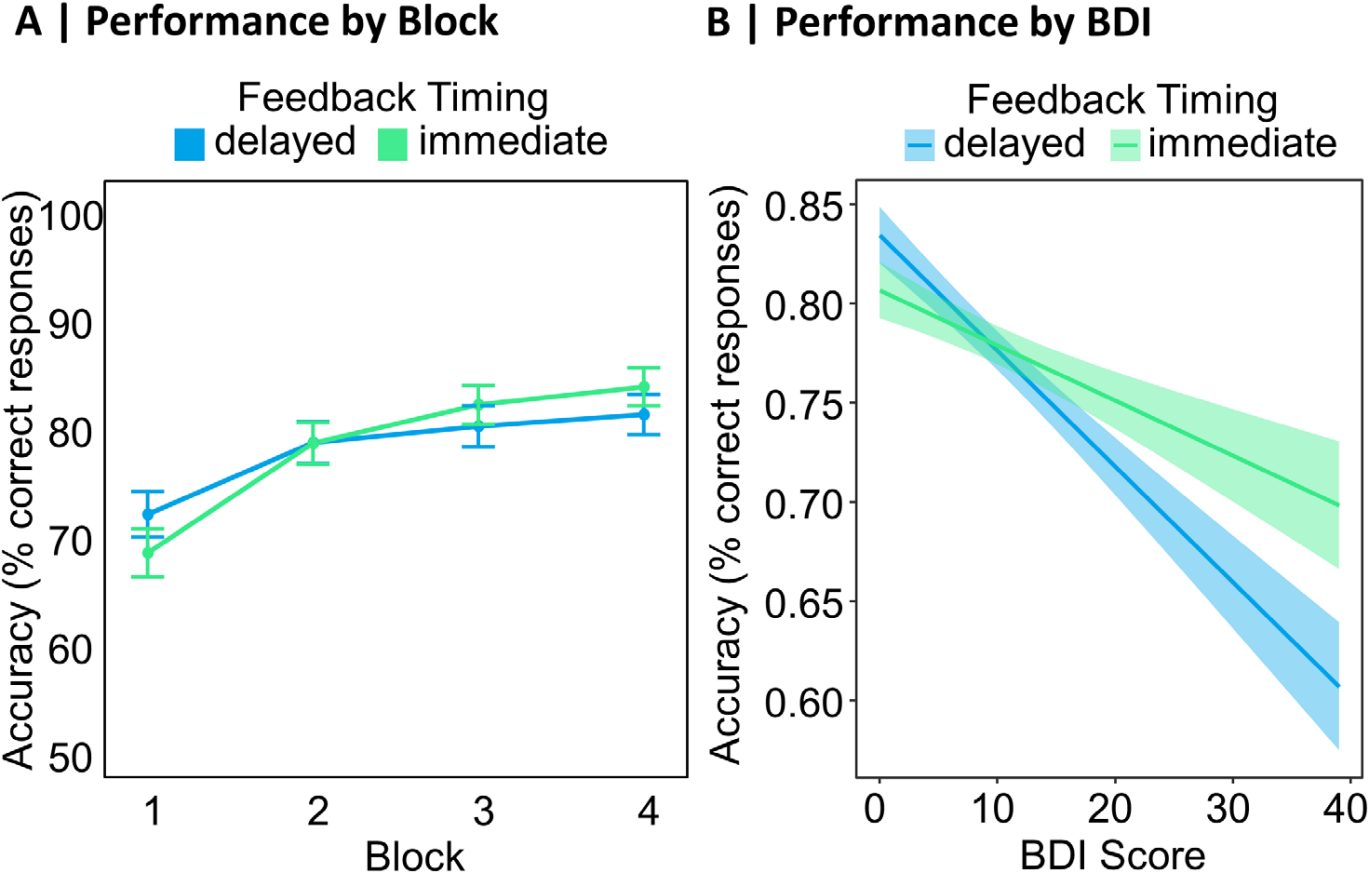
Descriptive pattern of learning performance during the feedback learning task. The plots are based on descriptive data. (A) Performance by Block: Mean accuracy (% of correct responses) for the four learning blocks of the probabilistic feedback learning task, separately for immediate and delayed feedback. Error bars represent 95% confidence intervals. (B) Performance by BDI: Mean accuracy (% of correct responses) in the probabilistic feedback learning task depending on BDI‐II Scores and Feedback Timing. Shaded areas represent 95% confidence intervals.

Table S5 in the Supporting Information lists *β*‐estimates and effect‐specific *z*‐tests for the three conducted GLME analyses, one for each depression measure (BDI, PHQ, and Familial Vulnerability). First, we report results from the GLME analysis including the BDI, together with Block and Feedback Timing. The analysis revealed a significant effect of Block (*p* < 0.001) on response accuracy, with an increasing number of correct responses (selection of the more frequently rewarded stimulus) across the four learning Blocks. Descriptive data for this effect are presented in Figure 2A. Furthermore, we found a significant effect of BDI (*p* = 0.011) in the direction that higher BDI scores led to a reduced performance in the experimental feedback learning task. Descriptive data underlying this effect are presented in Figure 2B. No other significant effects were observed (all *p*s ≥ 0.106). The models containing the PHQ and Familial Vulnerability both replicated the significant effect of Block (*p* < 0.001) found in the analysis including the BDI, while they did not reveal other significant effects (all *p*s ≥ 0.073 for the analysis involving PHQ and all *p*s ≥ 0.285 for the analysis involving Familial Vulnerability).

### 2.3 EEG Results

#### 2.3.1 FRN

**FIGURE 3:**
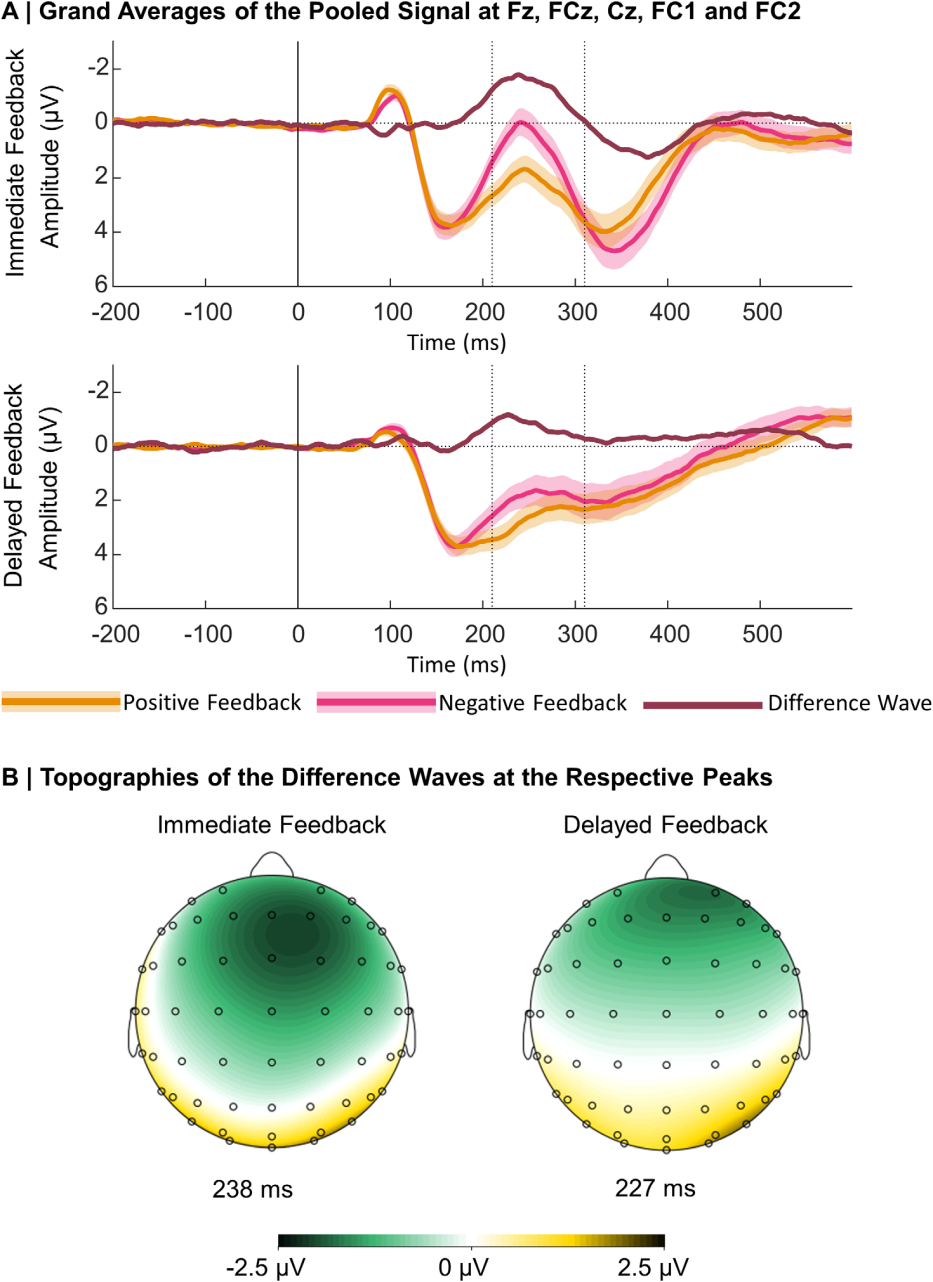
Grand Averages and topographical maps of the FRN. (A) Grand Averages: Dotted lines indicate the time window we used for the peak detection in the difference wave (negative—positive feedback). Shaded areas represent standard errors. (B) Topographies: The maps were constructed on the basis of the condition‐specific difference waves.

Grand averages for the ERPs following positive and negative immediate and delayed feedback pooled over the frontocentral cluster of electrodes are presented in Figure 3.

**FIGURE 4:**
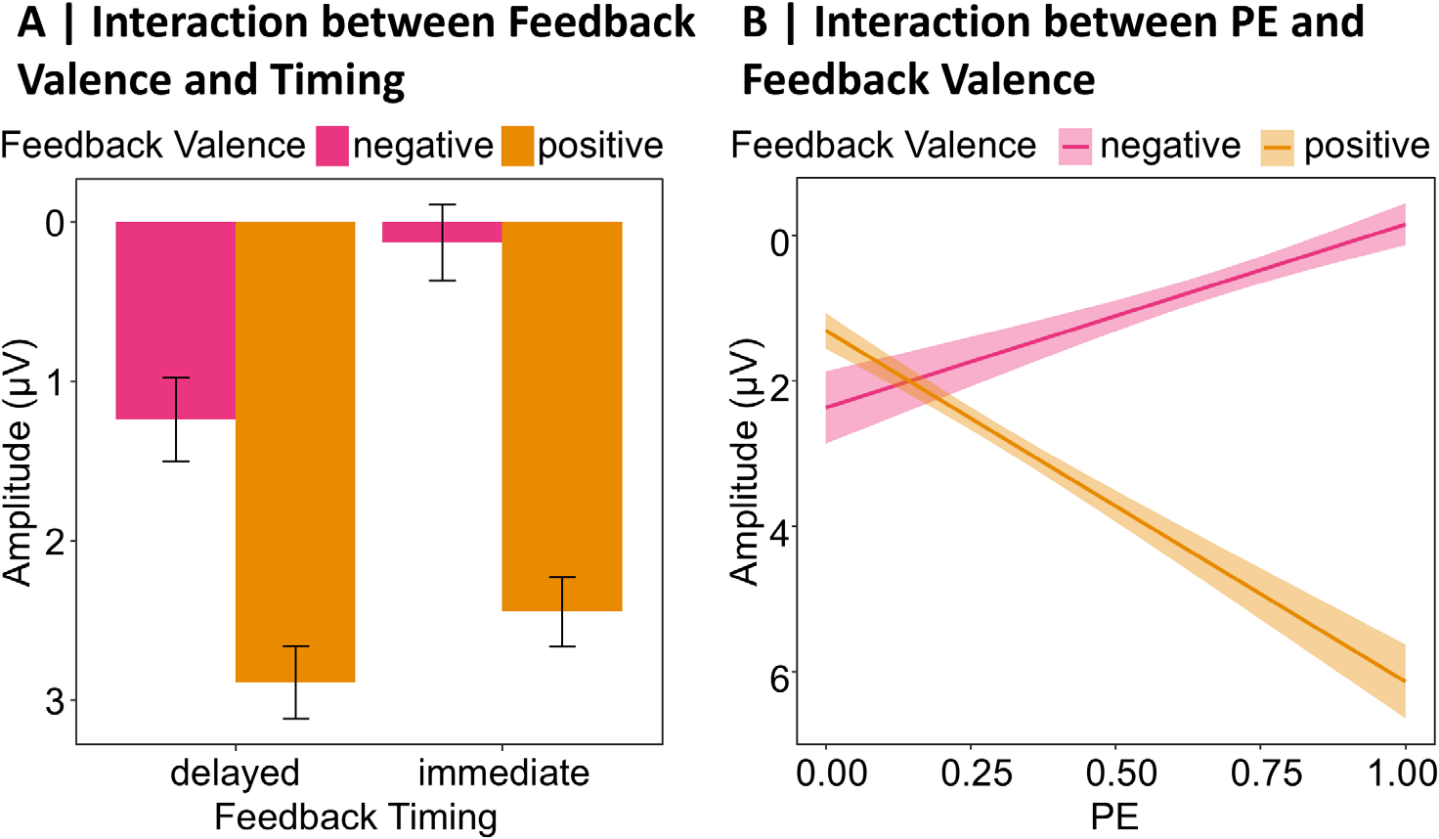
Descriptive data patterns underlying the FRN analyses. The plots are based on descriptive data. (A) Feedback Valence × Feedback Timing: *n* = 45. Error bars represent 95% confidence intervals. (B) PE × Feedback Valence: *n* = 45. Shaded areas represent 95% confidence intervals.

First, we begin by reporting the results of the LME analysis on the FRN, including BDI, Feedback Timing, Feedback Valence, and PE as predictors. Table S6 in the Supporting Information lists *β*‐estimates and effect specific *t*‐tests. The analysis revealed a significant effect of Feedback Valence (*p* < 0.001), with more negative amplitudes following negative compared to positive feedback. In addition, there was a significant effect of Feedback Timing (*p* = 0.004), indicating more negative amplitudes following immediate compared to delayed feedback. A significant interaction between Feedback Valence and Feedback Timing (*p* = 0.008) explained these effects further: Negative feedback was associated with more negative amplitudes for both immediate (*β* = 2.59, SE = 0.26, *t* = 9.85, *p* < 0.001) and delayed feedback (*β* = 1.85, SE = 0.27, *t* = 6.80, *p* < 0.001), but the effect was stronger for immediate feedback. Descriptive data underlying this interaction are presented in Figure 4A. Furthermore, we found a significant effect of PE (*p* = 0.008) that was further explained by a significant interaction between PE and Feedback Valence (*p* < 0.001), which we thus resolved. Descriptive data underlying this interaction are presented in Figure 4B. There was a significant effect of PE on the FRN amplitude for negative feedback, with more negative amplitudes for more unexpected feedback (*β* = −1.98, SE = 0.49, *t* = −4.01, *p* = 0.001). For positive feedback, this effect was reversed with more positive amplitudes for more unexpected feedback (*β* = 3.75, SE = 0.57, *t* = 6.56, *p* < 0.001). All other effects (including effects involving the BDI) were not significant (all *p*s ≥ 0.065).

Tables S7 and S8 in the Supporting Information list *β*‐estimates and effect specific *t*‐tests for the two models in which BDI was replaced by either PHQ or Familial Vulnerability. For both models, we could replicate the findings described for the BDI model—they are reported in detail in the Supporting Information under the sections titled FRN Analysis Including PHQ and FRN Analysis Including Familial Vulnerability, respectively. Apart from that, we did not find significant effects, neither of PHQ nor of Familial Vulnerability (all *p*s ≥ 0.271 for the model including PHQ and all *p*s ≥ 0.056 for the model including Familial Vulnerability).

#### 2.3.2 N170

**FIGURE 5:**
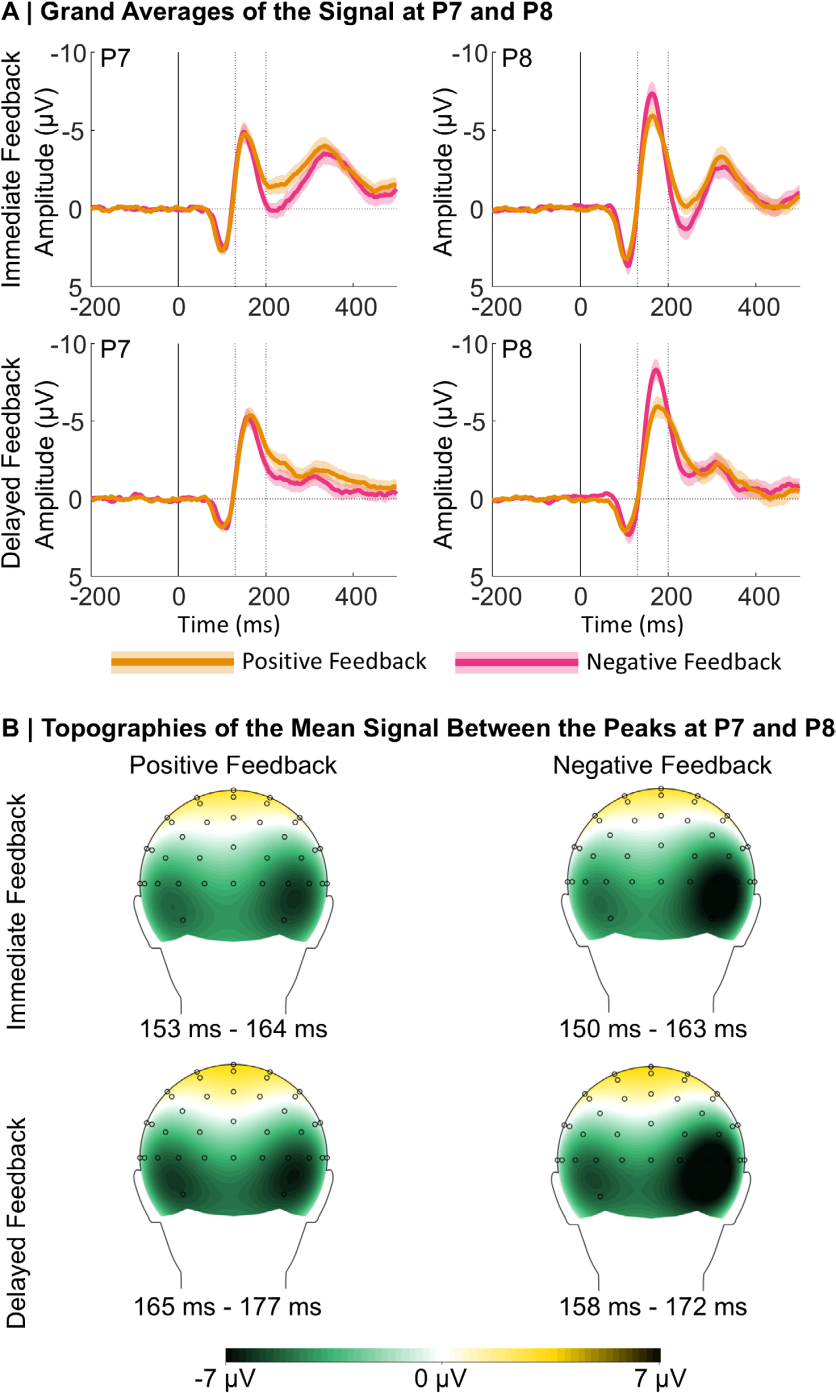
Grand averages at P7 and P8 and topographical maps at the respective peaks. (A) Grand Averages: Dotted lines indicate the time window we used for the N170 peak detection. Shaded areas represent standard errors. (B) Topographies: The maps were constructed on the basis of the condition‐specific N170 peaks.

Grand averages for the ERPs following positive and negative immediate and delayed feedback at electrode sites P7 and P8 are presented in Figure 5. In the following, we describe more negative N170 amplitudes as more pronounced or larger.

**FIGURE 6:**
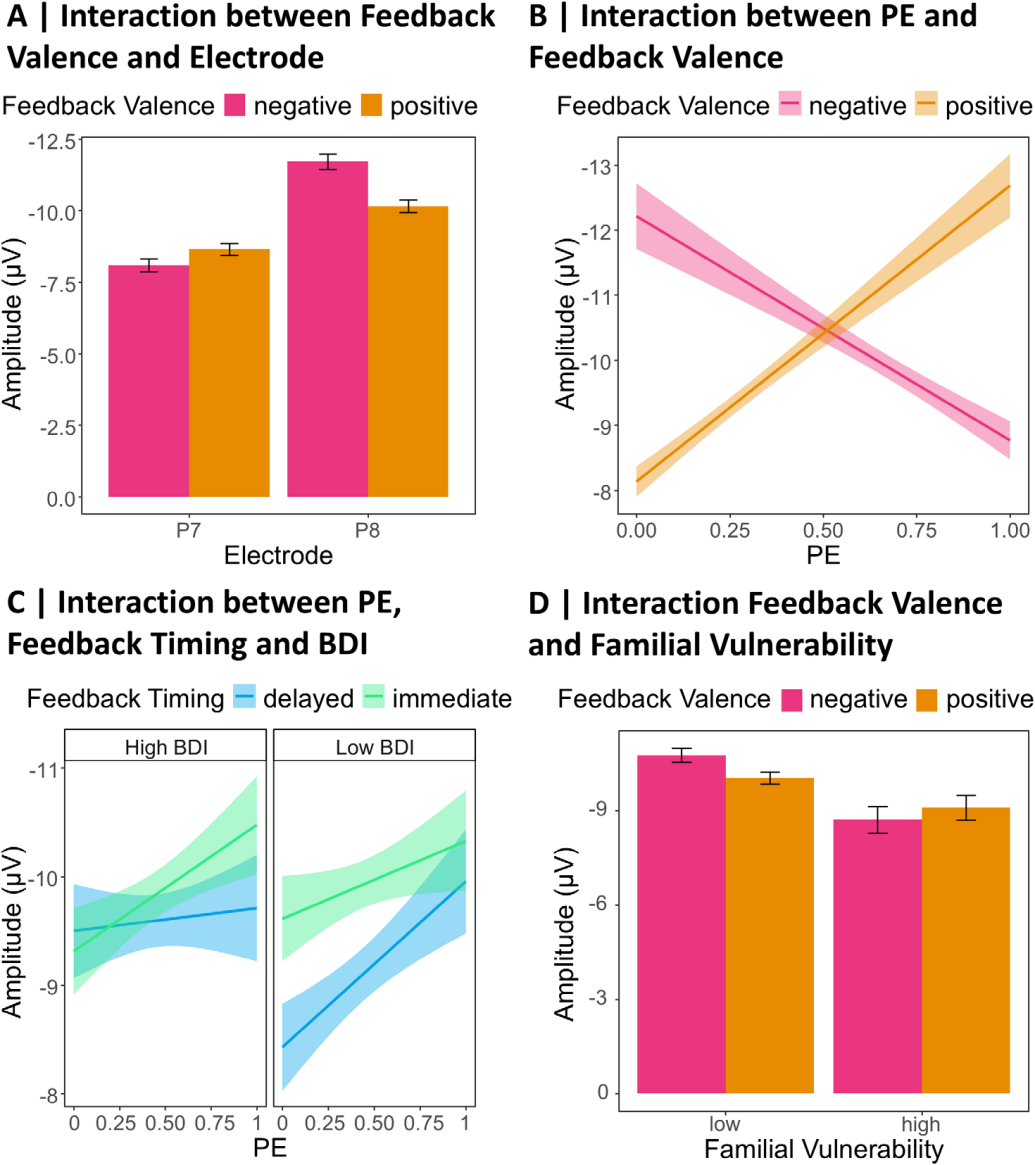
Descriptive data patterns underlying the N170 analyses. The plots are based on descriptive data. (A) Feedback Valence × Electrode: *n* = 45. Error bars represent 95% confidence intervals. (B) PE × Feedback Valence: *n* = 45. Shaded areas represent 95% confidence intervals. (C) PE × Feedback Timing × BDI: *n* = 45. The high BDI graph represents data from participants with BDI‐II scores ≥ median, the low BDI graph represents data from participants with BDI‐II scores < the median. Shaded areas represent 95% confidence intervals. (D) Feedback Valence × Familial Vulnerability: *n* = 37. Low Familial Vulnerability represents descriptive data from participants with first‐degree relatives without a history of depression, high Familial Vulnerability represents data from participants with first‐degree relatives with a depression diagnosis. Error bars represent 95% confidence intervals.

Similar to the FRN results, we first report results from the LME analysis on the N170 including the BDI, Feedback Timing, Feedback Valence, PE, and Electrode as predictors. Table S9 in the Supporting Information lists *β*‐estimates and effect specific *t*‐tests. The analysis revealed a significant main effect of the Electrode (*p* = 0.009), with more pronounced amplitudes over the right (P8) than the left hemisphere (P7). This main effect was further explained by a two‐way interaction between Electrode and Feedback Valence (*p* < 0.001), which we resolved with simple slope analyses. The underlying descriptive data are presented in Figure 6A. We found a significant effect of Feedback Valence only for P8 (*β* = 1.55, SE = 0.33, *t* = 4.67, *p* < 0.001) with larger amplitudes following negative compared to positive feedback, but not for P7 (*β* = −0.61, SE = 0.30, *t* = −2.02, *p* = 0.100). Furthermore, there was a significant interaction between Feedback Valence and PE (*p* < 0.001), which we resolved with simple slope analyses. The underlying descriptive data are presented in Figure 6B. For negative feedback, the PE had a significant effect on the N170, with more pronounced amplitudes for expected compared to unexpected feedback (*β* = 0.95, SE = 0.35, *t* = 2.69, *p* = 0.020). For positive feedback, the effect was reversed, with significantly larger N170 amplitudes for unexpected compared to expected feedback (*β* = −1.86, SE = 0.36, *t* = −5.17, *p* < 0.001).

**FIGURE 7:**
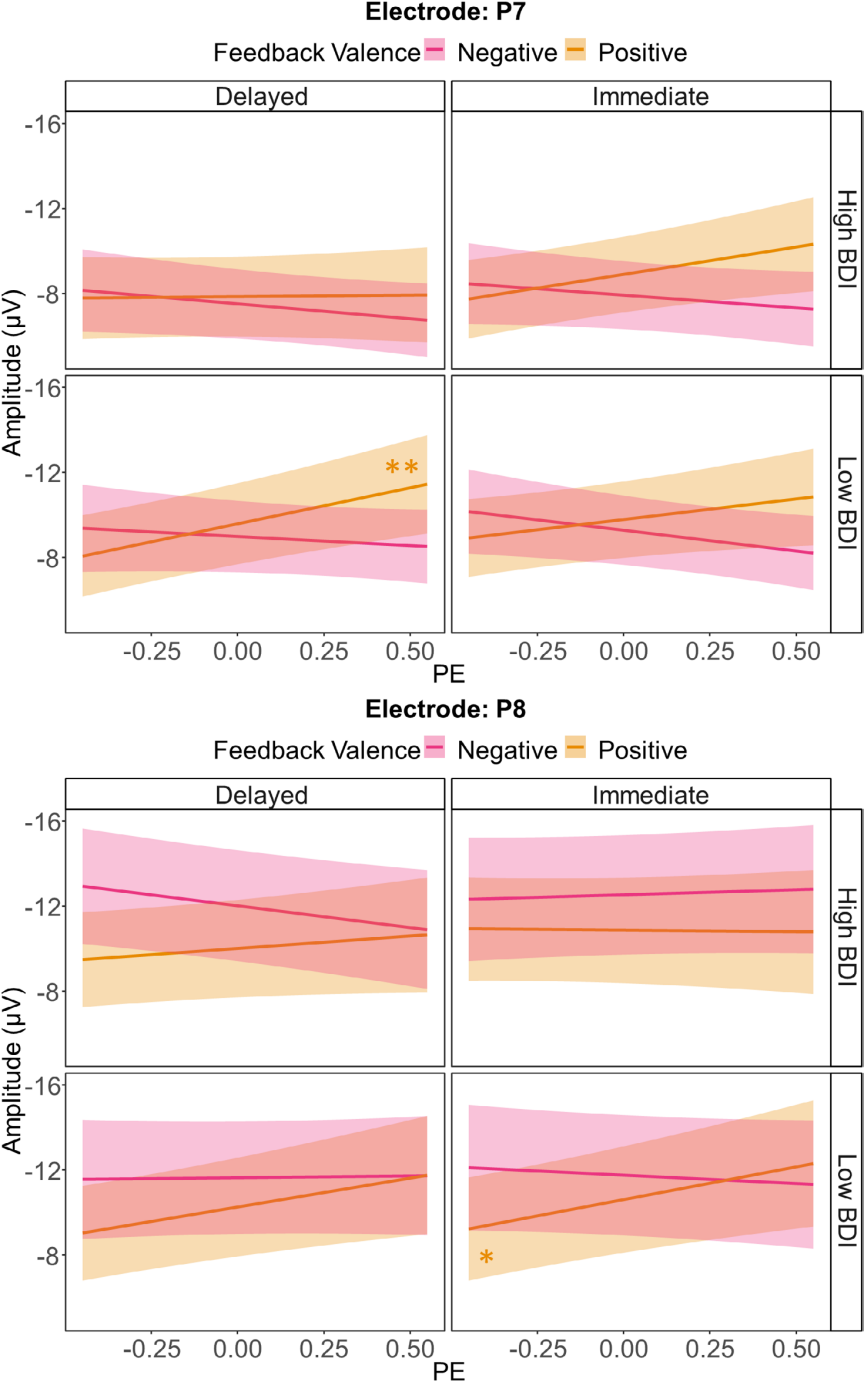
Model plots for the BDI × PE × Feedback Valence × Feedback Timing × Electrode interaction for the N170. Depicted are linear mixed‐effects model‐based predictions for the N170 amplitude at electrodes P7 and P8. Shaded areas indicate 95% confidence intervals. Stars indicate significance levels for the Bonferroni‐corrected *p*‐values that resulted from simple slopes analyses. *p < 0.050, **p < 0.010.

Regarding effects of the BDI, we found a significant three‐way interaction between BDI, Feedback Timing, and PE (*p* = 0.047). The underlying descriptive data are presented in Figure 6C. Simple slope analyses revealed that the PE only had a significant effect on the N170 following delayed feedback in participants with low BDI values (i.e., mean BDI‐1SD; *β* = −1.58, SE = 0.48, *t* = −3.30, *p* = 0.012). In individuals with no or only minimal depressive symptoms, the N170 amplitude increased for more unexpected delayed feedback. All other simple slope analyses did not reach significance (all *p*s ≥ 0.330; see Table S10 in the Supporting Information for *β*‐estimates and effect specific *t*‐tests). The three‐way interaction was further explained by a significant five‐way interaction between all predictors included in the analysis (*p* = 0.041), which we resolved using simple slope analyses. Model plots for P7 and P8 separately are presented in Figure 7, Table S11 in the Supporting Information lists *β*‐estimates and effect specific *t*‐tests. The simple slope analyses revealed a significant effect of the PE on the N170 for the P7 in participants with low BDI scores after receiving delayed positive feedback (*β* = −3.36, SE = 0.92, *t* = −3.67, *p* = 0.004), with larger amplitudes for more unexpected feedback. For the P8, the simple slope analysis also revealed a significant PE effect on the N170, again in participants with low BDI scores and following positive feedback, but this time for immediate feedback (*β* = −3.09, SE = 1.00, *t* = −3.09, *p* = 0.034). Again, more unexpected positive feedback was associated with larger N170 amplitudes. Apart from these two significant slopes, the simple slope analyses did not reveal any further significant effects (all *p*s ≥ 0.051). All other main and interaction effects of the LME analysis including the BDI did not reach significance (all *p*s ≥ 0.063).

In summary, two key results emerged for the N170. First, this ERP component is sensitive to feedback valence—particularly over the right hemisphere, where losses elicit larger amplitudes than gains—but this effect is absent in participants with a familial history of depression. Second, when considering PE processing in the N170 separately for each condition and in participants scoring low and high on depressive symptoms, representations of (positive) PEs are only found in participants with low depressive symptoms.

For the analysis containing the PHQ instead of the BDI as a predictor, Table S12 in the Supporting Information lists *β*‐estimates and effect specific *t*‐tests. To avoid redundancies, we only focus on effects involving the PHQ, while other effects (mainly replications of effects that were already described for the BDI analysis) are reported in the section titled N170 Analysis Including PHQ of the Supporting Information. The analysis revealed neither a significant main effect of PHQ, nor significant interactions involving PHQ (all *p*s ≥ 0.054).

For the analysis containing Familial Vulnerability, Table S13 in the Supporting Information lists *β*‐estimates and effect specific *t*‐tests. As above, here we only report effects involving Familial Vulnerability, while other effects (again mainly replications already described for the BDI model above) are reported in the Supporting Information under the section titled N170 Analysis Including Familial Vulnerability. The analysis revealed a significant interaction between Feedback Valence and Familial Vulnerability (*p* = 0.011). The underlying descriptive data are presented in Figure 6D. Only participants without first‐degree relatives with a history of depression showed significantly larger amplitudes for negative compared to positive feedback (*β* = 0.93, SE = 0.29, *t* = 3.20, *p* = 0.006), while there was no effect of Feedback Valence for participants with first‐degree relatives with a diagnosed depression (*β* = −0.74, SE = 0.54, *t* = −1.38, *p* = 0.358). Apart from that, there were no other significant effects involving Familial Vulnerability (all *p*s ≥ 0.064).

## 3 Discussion

The present study aimed to investigate links between depressive symptoms and feedback learning and feedback processing using insights from two ERP components, FRN and N170, that have primarily been associated with immediate and delayed feedback processing, respectively. Previous studies reported a reduced differentiation between responses to gains and losses in FRN amplitudes in combination with reduced striatal processing in depression (Bress et al. 2012, 2015; Foti et al. 2014; Klawohn et al. 2021; Pizzagalli et al. 2009; Takamura et al. 2017; for reviews see Admon and Pizzagalli 2015, Keren et al. 2018 and Luking et al. 2016). However, the striatum seems to be particularly important for immediate feedback processing, while for delayed feedback processing hippocampal activity is increased, which has been linked to the N170 ERP component (Arbel et al. 2017; Foerde et al. 2013; Foerde and Shohamy 2011; Höltje and Mecklinger 2020; Kim and Arbel 2019; Peterburs et al. 2016; Weinberg et al. 2012; Weismüller and Bellebaum 2016). This is the first study to explicitly address effects of depressive symptoms on learning from and processing of immediate and delayed feedback. Besides trying to replicate findings of impaired learning (Admon et al. 2017; Bakic et al. 2017; Kumar et al. 2018; Kunisato et al. 2012; Macoveanu et al. 2014; Pechtel et al. 2013; Pizzagalli et al. 2005, 2008) and reduced differentiations between positive and negative feedback in FRN amplitudes following immediate feedback in depression (Bress et al. 2012, 2015; Foti et al. 2014; Klawohn et al. 2021), we hypothesized to find alterations for learning from delayed feedback and also delayed feedback processing as measured via the N170. As hypothesized, we found that learning performance decreased with more severe currently experienced depressive symptoms, irrespective of feedback delay. While we could not replicate findings of a reduced differentiation between responses to gains and losses in FRN amplitude for more severe depressive symptoms, we found PE coding in the N170 only in participants with low BDI scores, mainly driven by responses to (delayed) positive feedback. In addition, familial vulnerability for depression was linked to a reduced sensitivity for feedback valence encoded in the N170.

### 3.1 Effects of Current Depressive Symptoms on Feedback Learning, FRN and N170

The ability to use positive and negative consequences of our actions to learn and shape future decisions is fundamental for human intelligent behavior (Silver et al. 2021). Accordingly, participants in our study successfully learned to choose the more rewarding out of two stimuli throughout a probabilistic feedback learning task that required an accumulation of experiences over time (Fu and Anderson 2008). Previous studies indicated that depression interferes with the ability to learn from feedback (Admon et al. 2017; Bakic et al. 2017; Kumar et al. 2018; Kunisato et al. 2012; Macoveanu et al. 2014; Pechtel et al. 2013; Pizzagalli et al. 2005, 2008; for reviews see Chen et al. 2015 and Eshel and Roiser 2010). In line with this and our hypothesis, we found that participants currently experiencing more severe depressive symptoms showed worse learning performance in the probabilistic feedback learning task, regardless of feedback timing. This is in line with our assumption that both learning from immediate and delayed feedback are affected by depression, possibly caused by (functional) changes in the striatum and hippocampus (Admon and Pizzagalli 2015; Fairhall et al. 2010; Luking et al. 2016; Nestler et al. 2002; Pizzagalli et al. 2009; Takamura et al. 2017; Thompson 2023) that are both involved in feedback processing (Foerde et al. 2013; Foerde and Shohamy 2011).

However, these behavioral alterations were not reflected in the FRN: Against our hypothesis, we did not find reduced feedback valence sensitivity in FRN amplitudes in participants currently experiencing depressive symptoms, which is not in line with previous findings (Bress et al. 2012, 2015; Foti et al. 2014; Klawohn et al. 2021). An explorative investigation revealed that PE coding in the FRN was also not affected by the severity of currently experienced depressive symptoms. In contrast, a study by Jackson and Cavanagh (2023) indicated that low mood can influence reward learning via poorer PE coding in the RewP. On the one hand, our results seem to support studies that found no or only weak, task‐dependent relationships between the FRN and depression (Hager et al. 2021; Clayson et al. 2020; Moran et al. 2017). On the other hand, the EEG signal is influenced by many cognitive processes, and their spatiotemporal overlaps can make it difficult to link individual performance to ERP deflections (Ullsperger 2024). Thus, dissociations between the FRN and behavior are not an uncommon finding (Ullsperger 2024), indicating that other neural processes, reflected in other feedback‐locked ERP components, might be more closely linked to behavior.

The present study is the first to investigate depression‐related alterations in the feedback‐locked N170. Previous studies found more pronounced N170 amplitudes following delayed feedback compared to immediate feedback (Arbel et al. 2017; Höltje and Mecklinger 2020; Kim and Arbel 2019). Because of the role of the hippocampus for delayed feedback processing (Foerde et al. 2013; Foerde and Shohamy 2011), the N170 has been interpreted to reflect MTL activity (Arbel et al. 2017; Höltje and Mecklinger 2020; Kim and Arbel 2019). Depression can be accompanied by changes in hippocampal structure and functioning, possibly explaining memory impairments and some of the cognitive symptoms seen in depression (Fairhall et al. 2010; Nestler et al. 2002; Thompson 2023). Therefore, we expected reduced N170 amplitudes for participants currently experiencing more severe depressive symptoms, especially following delayed feedback. While we could not find the hypothesized pattern, currently experienced depressive symptoms affected PE coding reflected in the N170. More specifically, we found reflections of the PE in the N170 amplitude only in participants with low levels of depressive symptoms and especially following (delayed) positive feedback. For these participants, more unexpected positive feedback was linked to more pronounced N170 amplitudes. While this is not exactly what we had hypothesized, it still matches our assumption that structures and processes that are involved in generating the N170 are altered in depression.

For the feedback‐locked N170, it was only twice previously described that it reflects the entire range of the PE (Röhlinger, Albrecht, and Bellebaum 2025; Röhlinger, Albrecht, Ghio, et al. 2025), with an opposite pattern compared to the FRN (Burnside et al. 2019; Fischer and Ullsperger 2013; Weber and Bellebaum 2024): the more unexpected positive feedback was, the more negative (pronounced) the N170 became and the more unexpected negative feedback was, the more positive it became. In the present study we could replicate this finding. Since midbrain dopamine neurons seem to send information not only to striatal and fronto‐cortical areas of the brain (Schultz 2002), but also to the hippocampus (Calabresi et al. 2013; Tsetsenis et al. 2023), the described pattern of particularly pronounced N170 amplitudes for unexpected gains could mean that the MTL is especially involved in reactivating representations of unexpectedly rewarded stimuli to link them to (temporally delayed) feedback (Röhlinger, Albrecht, and Bellebaum 2025). In contrast, the particularly small N170 amplitudes for unexpected losses seem more difficult to interpret. On the other hand, ERP amplitudes cannot directly be linked to more or less neural activity in particular brain regions (Luck 2014). Thus, the crucial point to be emphasized is that the N170 reflects a neural signature of PEs in addition to the known signature in the FRN/RewP. While in healthy individuals, midbrain dopamine regions that encode PEs possibly interact with the MTL to enhance memory representations of rewarded stimuli to adapt future behavior (Shohamy and Adcock 2010), our results suggest that this process might be disrupted by acute depression. This would be in line with findings of hippocampal atrophy and changes in cognitive functions like memory (Fairhall et al. 2010; Nestler et al. 2002; Thompson 2023). In addition, acute depressive symptoms come along with anhedonia, reduced reward responsiveness and altered feedback learning processes (Admon et al. 2017; Bakic et al. 2017; Heshmati and Russo 2015; Huys et al. 2013; Kumar et al. 2018; Kunisato et al. 2012; Rizvi et al. 2016), which might be related to the reduced PE coding following positive feedback in the present study.

However, structures beyond the dopaminergic midbrain also respond to unexpected events, including rewards. For instance, PE signals originating from the norepinephrine‐releasing locus coeruleus may influence N170 amplitudes through its projections to the hippocampus (Sara 2009, 2015; Schultz and Dickinson 2000).

### 3.2 Effects of Past Depressive Episodes on Feedback Learning, FRN, and N170

Since individuals who have recovered from depression still show blunted responses to reward in the striatum (McCabe et al. 2009), we intended to not only look at effects of currently experienced depressive symptoms, but also investigate effects of past depressive symptom severity on feedback learning and processing, with a new focus on delayed feedback processing and potentially reduced N170 amplitudes. However, we did not find any effects of past depressive episodes as measured via a modified version of the PHQ‐9. The modified version of the PHQ‐9 was introduced as a measure of lifetime depression (Cannon et al. 2007) in which participants rate how strongly they have experienced a list of depressive symptoms in the 2 weeks in their life in which they have felt most sad or depressed. However, scoring high on this questionnaire does not necessarily indicate that a person went through a major depressive episode in the past. For example, losing a beloved person can cause a feeling of sadness or emptiness, a loss of pleasure or interest in activities, or one of the other symptoms that are among the diagnostic criteria for a major depressive episode according to the International Classification of Diseases (ICD‐11, World Health Organization 2019) and that are assessed by the modified PHQ‐9. Apparently, there is an overlap of symptoms between grief and depression and although grief can culminate in a major depression, it is usually not pathological (Shear et al. 2011; Zisook and Shear 2009). We assume that the modified PHQ‐9 used in this study might have been unsuitable to reliably and validly assess clinically relevant past depressive episodes.

### 3.3 Effects of Familial Vulnerability on Feedback Learning, FRN, and N170

A familial history of depression, for example having a depressed mother, poses a risk of developing depression (Halligan et al. 2007; Raposa et al. 2014). Accordingly, blunted FRN amplitudes following rewards were found in siblings of depressed individuals who were not depressed themselves (Weinberg et al. 2015). Blunted responses to reward within the dorsal and ventral striatum relative to children of nondepressed mothers serve as a neurophysiological explanation for the increased risk and altered FRN amplitudes (for an extensive review see Luking et al. 2016). We intended to find out whether familial vulnerability for depression, besides affecting the FRN following immediate feedback, also affects the processing of delayed feedback, for example indicated by reduced N170 amplitudes. First of all, familial vulnerability did not affect behavioral response accuracy in the probabilistic feedback learning task and we did not find vulnerability‐related changes in the FRN in terms of a reduced sensitivity for feedback valence. However, we observed such an effect for the N170, independent of feedback timing: In general, the N170 in this study was more pronounced following negative than positive feedback (for similar results see Kim and Arbel 2019), but only over the right hemisphere. This sensitivity for feedback valence was not found in participants with a familial history of depression. In other words, the amplitude difference between positive and negative feedback in the N170 diminished for participants at increased risk for depression. In this line, an fMRI study on 10‐ to 14‐year‐old girls with a familial history of depression found reduced striatal activity in response to rewards and increased activation in the dorsal anterior cingulate cortex following losses (compared to peers without such a family history) even before the onset of depressive symptoms (Gotlib et al. 2010). Similar alterations in the activity of structures underlying the N170 could account for the altered processing of positive and negative feedback observed in this study.

### 3.4 Limitations and Future Studies

While it seems to be justified to consider depression not as a dichotomous variable, there are also limitations of this approach. The participants in our study reached a mean BDI score of 9.89 (SD = 9.34), which is below the cut‐off score for depression (von Glischinski et al. 2019). Some participants scored really low (Min = 0) and the maximum score was 39 in our sample (with respect to a theoretically possible score of 63), indicating that the variance in currently experienced depressive symptomatology was limited. Even though Bress et al. (2012, 2015) found correlations between the FRN amplitude and self‐rated depressive symptoms in a nonclinical sample, their sample cannot be compared to ours, as it consisted of 8‐ to 13‐year‐old children/adolescents, and depression‐related alterations in the FRN seem to be most pronounced in individuals under age 18 (Keren et al. 2018). A lack of participants with very high BDI scores may have prevented us from finding such a relationship in an adult sample (Clayson et al. 2020). In addition, self‐reported depression assessed via the BDI‐II in our mostly undergraduate academic participants may have been confounded by academic or peer‐related stressors and therefore less indicative of a major depressive disorder (Hager et al. 2021). On the contrary, the variance in BDI scores in our sample was sufficient to detect effects on behavioral response accuracy and the N170. Other studies that found effects of depression on the FRN compared clinical samples, i.e., participants that met the criteria for a clinical diagnosis of unipolar depression (and reached a BDI score ≥ 13), with healthy controls (Foti et al. 2014; Klawohn et al. 2021). Future studies could try to overcome variance issues by combining group designs (clinical vs. nonclinical sample) with the inclusion of depression as a continuous variable in the analysis.

Another sample‐related limitation of our results involves the prevalence of other psychological disorders apart from depression, which were identified by the Mini‐DIPS. For example, 26% of our participants met the criteria for a social anxiety disorder (currently or in the past). Heightened responses to negative feedback in the FRN have been observed in anxiety (Tobias and Ito 2021), which may blur the effects of depressive symptoms. Grabowska et al. (2024) explain that due to evidence for ERP components being modulated by various interindividual differences, focusing on a small set of them might be problematic: Psychological disorders like anxiety and depression are interconnected and their influence on an individual's way of processing feedback may be either direct or indirect. Furthermore, depression encompasses a wide variety of emotional, cognitive, behavioral, and neurovegetative symptoms (see ICD‐11, World Health Organization 2019), and understanding the complex relationships between them and feedback processing requires further investigation. For example, it is possible that only a specific subtype of depression—particularly characterized by anhedonia—exhibits alterations at the level of the FRN. Finally, heterogeneity in the etiology of depression (Kendler et al. 2002) may account for the difficulties of replicating effects on feedback processing. Grabowska et al. (2024) suggest tackling the challenges in research on relationships between ERP components and psychopathologies with network analysis techniques. Despite the limitations associated with the BDI and the heterogeneity of the sample, the FRN in our study was sensitive to both feedback valence and PEs but not to depression, representing an important null finding (for similar results, see Hager et al. 2021; Clayson et al. 2020; Moran et al. 2017).

With regard to the effects of acutely experienced depressive symptoms as well as familial vulnerability to depression on the N170, it should be noted as a limitation that the analyses employed were highly complex, while the sample size, with 45/37 participants, was relatively small. In addition, the PE modeling yielded very low estimated learning rates and/or exploration parameters near the upper bound for some participants (see Figure S2). This is not unusual and may reflect poor behavioral performance in these participants. Conversely, such boundary estimates may also indicate limitations of the estimation process. In particular, exploration parameters above 10 become functionally very similar, indicating near‐deterministic exploitation, which makes their precise estimation challenging. Accordingly, these values should be interpreted carefully and not as exact point estimates. In summary, it is essential to examine whether the findings reported here can be replicated.

## 4 Conclusion

In the present study, we found that performance in a learning task decreased with more severe depressive symptoms, for learning from both immediate and delayed feedback. While the FRN was unaffected by acute depressive symptom severity, past depressive episodes, and familial vulnerability for depression, we found depression‐related changes in the N170, remarkably for both immediate and delayed feedback processing. Currently experienced depressive symptoms were associated with poorer encoding of prediction errors in the N170, possibly explaining reduced learning performance. In addition, a family history of depression was associated with reduced sensitivity to feedback valence in the N170. Thus, the N170 emerges as a novel, important biomarker alongside the FRN in clinical research on depression and feedback‐based learning processes.

## Author Contributions

**Madita Röhlinger:** conceptualization, data curation, formal analysis, investigation, methodology, project administration, validation, visualization, writing – original draft, writing – review and editing. **Julian Vahedi:** validation, visualization, writing – review and editing. **Christian Bellebaum:** conceptualization, methodology, project administration, resources, supervision, validation, writing – review and editing.

## Funding

This research did not receive any specific grant from funding agencies in the public, commercial, or not‐for‐profit sectors.

## Conflicts of Interest

The authors declare no conflicts of interest.

## Supporting information


**Data S1:** psyp70228‐sup‐0001‐Supinfo.docx.

## Data Availability

The data that support the findings of this study are openly available in the Open Science Framework at https://osf.io/qu7td/.
