# Supplementary material for: Links Between Altered Feedback Learning and Symptoms of Depression: Insights From the FRN and Feedback‐Locked N170
Source: Psychophysiology. 2026 Jan 9;63(1):e70228. doi: 10.1111/psyp.70228 (PMC12789050; doi:10.1111/psyp.70228)
Supplement: Supplementary file 1 — Data S1: psyp70228‐sup‐0001‐Supinfo.docx. [file PSYP-63-e70228-s001.docx]

Supporting Information

Links between Altered Feedback Learning and Symptoms of Depression: Insights from the FRN and feedback-locked N170

Madita Röhlinger, Julian Vahedi & Christian Bellebaum

**Figure S1**


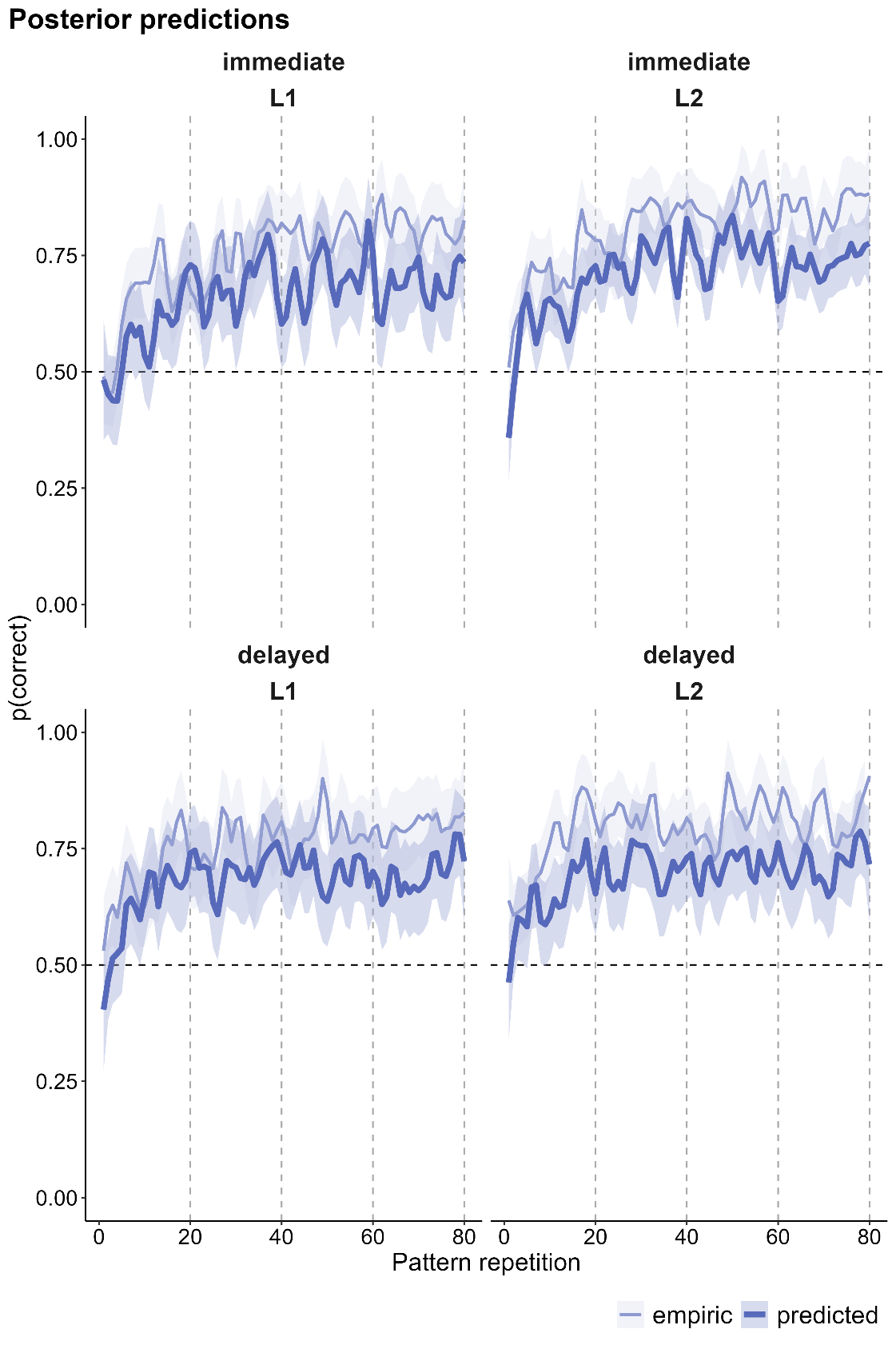


*Note.* Group-level posterior predictive check. Thinner, lighter lines represent observed trial-by-trial empirical choice accuracy, whereas thicker, darker lines represent choice accuracy predicted by the reinforcement learning model based on simulated data. Results are shown separately for each feedback timing condition and learning phase, and are presented as mean ± standard error. The reinforcement learning model generally captures the overall trajectory of the empirical data, but slightly underpredicts choice accuracy, particularly in the second half of each learning phase. This pattern is consistent across both feedback timing conditions and learning phases. Abbreviations: L1 = first learning phase; L2 = second learning phase.

**Figure S2**


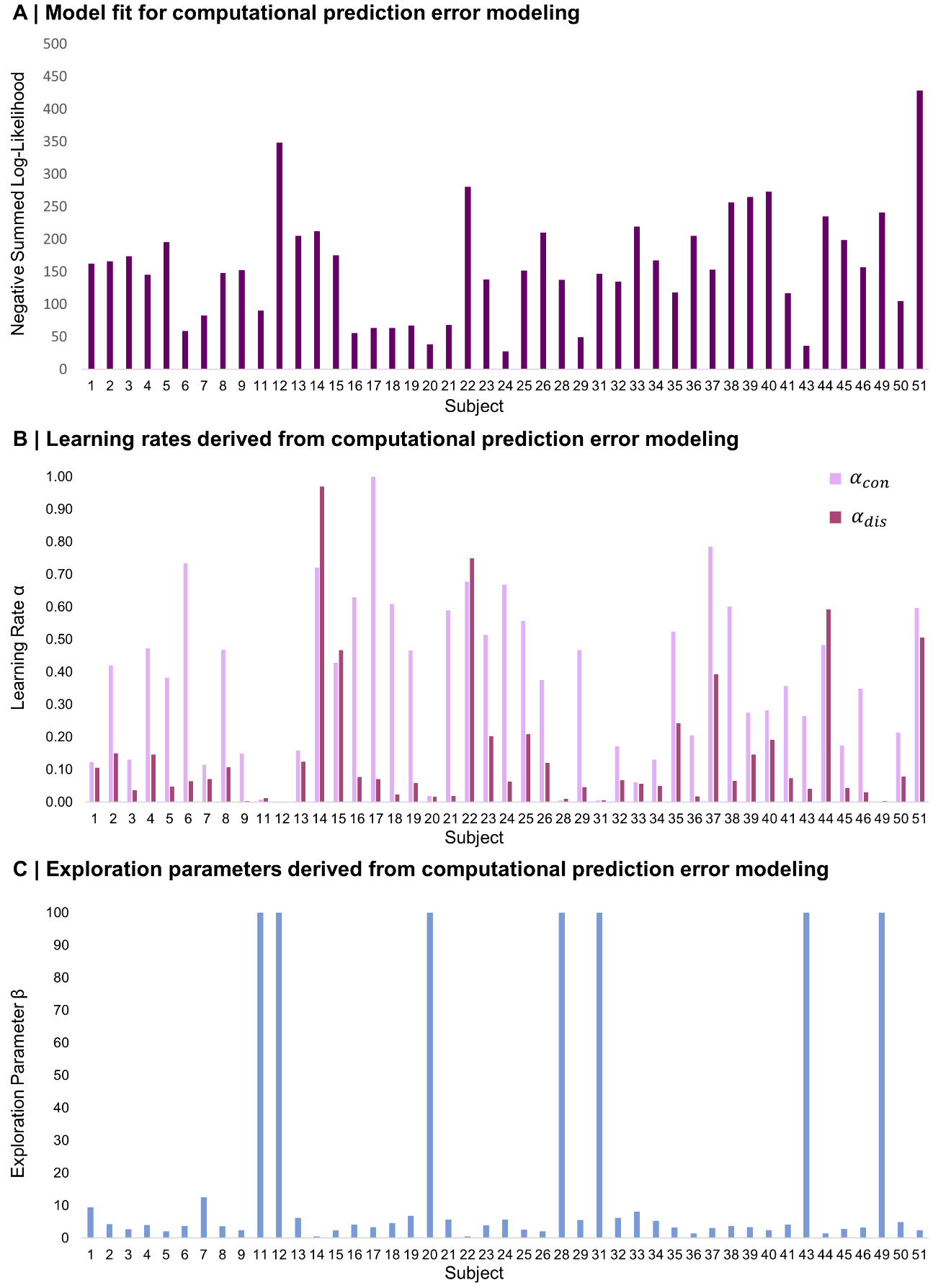


*Note.* **A** Model fit: $-LL$= negative summed log-likelihood as measure for the model’s goodness of fit per subject across conditions. **B** Learning Rates: ­$\alpha_{con}$ = estimated learning rate from positive feedback that confirms the choice, ­$\alpha_{dis}$ = estimated learning rate from negative feedback that disconfirms the choice. C Exploration Parameter: higher $\beta$ indicates reliance on previous stimulus values, small $\beta$ indicates explorative choice behavior.

| **Table S1** | |
| --- | --- |
| *Maximal GLME models for the analysis of behavioral data* | |
| Model | Formula |
| Current depressive symptoms | *Accuracy ~ 1 + Block + BDI + Block:BDI + Timing + BDI:Timing + Block:Timing + Block:BDI:Timing + (1 + Block + Timing + Timing:Block \| Participant)* |
|  |  |
| Past depressive episodes | *Accuracy ~ 1 + Block + PHQ + Block:PHQ + Timing + Block:Timing + PHQ:Timing + Block:PHQ:Timing + (1 + Block + Timing + Timing:Block \| Participant)* |
|  |  |
| Familial vulnerability | *Accuracy ~ 1 + Block + Vulnerability + Timing + Block:Timing + Vulnerability:Timing + Block:Vulnerability + Block:Vulnerability:Timing +(1 + Block + Timing + Timing:Block \| Participant)* |
| *Note.* GLME = generalized linear mixed effects. BDI = BDI-II (mean centered, yielding negative values for scores below the mean vs. positive values for scores above the mean), PHQ = modified PHQ-9 (mean centered, yielding negative values for scores below the mean vs. positive values for scores above the mean), Vulnerability = Familial Vulnerability (first-degree relatives without a history of depression [−0.5] vs. first-degree relatives with a depression diagnosis [0.5]), Timing = Feedback Timing (immediate [-0.5] vs. delayed [0.5]). | |

| **Table S2** | | | | | | | | |
| --- | --- | --- | --- | --- | --- | --- | --- | --- |
| *Correlations between latent variables from prediction error modeling and depression variables* | | | | | | | | |
|  |  | β | | α_con_ | | α_dis_ | |  |
|  | *n* | *r* | *p* | *r* | *p* | *r* | *p* |  |
| BDI | 45 | -.12 | .444 | -.04 | .793 | .09 | .538 |  |
| PHQ | 45 | .05 | .745 | .02 | .907 | .08 | .596 |  |
| Family | 37 | -.06 | .722 | .10 | .571 | .17 | .321 |  |
| *Note.* BDI = Beck Depression Inventory II, PHQ = Mood Module of the Patient Health Questionnaire, Family = categorical variable indicating whether a first-degree relative has ever been diagnosed with depression (0 = no familial vulnerability, 1 = familial vulnerability), β = exploration parameter in the probabilistic feedback learning task, α_con_ = learning rate in trials with positive feedback, α_dis_ = learning rate in trials with negative feedback, r = pearson correlation coefficient. | | | | | | | | |

| **Table S3** | |
| --- | --- |
| *Maximal LME models for the FRN analysis* | |
| Model | Formula |
| Current depressive symptoms (BDI-II) | *FRN Amplitdue ~ 1 + Timing + Valence + Timing:Valence + BDI + Timing:BDI + Valence:BDI + Timing:Valence:BDI + PE + Timing:PE + Valence:PE + Timing:Valence:PE + BDI:PE + Timing:BDI:PE + Valence:BDI:PE + Timing:Valence:BDI:PE + (1 + Timing + Valence + PE + Valence:PE \| Participant)* |
| Past depressive episodes (PHQ) | *FRN Amplitdue ~ 1 + Timing + Valence + Timing:Valence + PHQ + Timing:PHQ + Valence:PHQ + Timing:Valence:PHQ + PE + Timing:PE + Valence:PE + Timing:Valence:PE + PHQ:PE + Timing:PHQ:PE + Valence:PHQ:PE + Timing:Valence:PHQ:PE + (1 + Timing + Valence + PE + Valence:PE \| Participant)* |
|  |  |
| Familial vulnerability | *FRN amplitude ~ 1 + Timing + Valence + Timing:Valence + Vulnerability + Timing:Vulnerability + Valence:Vulnerability + Timing:Valence:Vulnerability + PE + Timing:PE + Valence:PE + Timing:Valence:PE + Vulnerability:PE + Timing:Vulnerability:PE + Valence:Vulnerability:PE + Timing:Valence:Vulnerability:PE + (1 + Timing + Valence + PE \| Participant)* |
| *Note.* LME = linear mixed effects. BDI = BDI-II (mean centered, yielding negative values for scores below the mean vs. positive values for scores above the mean), PHQ = modified PHQ-9 ( mean centered, yielding negative values for scores below the mean vs. positive values for scores above the mean), Vulnerability = Familial Vulnerability (first-degree relatives without a history of depression [−0.5] vs. first-degree relatives with a depression diagnosis [0.5]), Timing = Feedback Timing (immediate [-0.5] vs. delayed [0.5]), Valence = Feedback Valence (negative [-0.5] vs. positive [0.5]), PE = unsigned PE (scaled and mean centered, yielding negative values for PE values below the mean vs. positive values for PE values above the mean). | |

| **Table S4** | |
| --- | --- |
| *Maximal LME models for the N170 analysis* | |
| Model | Formula |
| Current depressive symptoms (BDI-II) | *N170 Amplitude ~ 1 + Timing + Valence + Timing:Valence +*  *BDI + Timing:BDI + Valence:BDI + Timing:Valence:BDI + PE + Timing:PE + Valence:PE + Timing:Valence:PE + BDI:PE + Timing:BDI:PE + Valence:BDI:PE + Timing:Valence:BDI:PE + Electrode + Timing:Electrode + Valence:Electrode + Timing:Valence:Electrode + BDI:Electrode + Timing:BDI:Electrode + Valence:BDI:Electrode + Timing:Valence:BDI:Electrode + PE:Electrode + Timing:PE:Electrode + Valence:PE:Electrode + Timing:Valence:PE:Electrode + BDI:PE:Electrode + Timing:BDI:PE:Electrode + Valence:BDI:PE:Electrode + Timing:Valence:BDI:PE:Electrode + (1 + Electrode + Timing + Valence + Valence:Electrode + Timing:Electrode + PE \| Participant)* |
| Past depressive episodes (modified PHQ) | *N170 amplitude ~ 1 + Timing + Valence + Timing:Valence + PHQ + Timing:PHQ + Valence:PHQ + Timing:Valence:PHQ + PE + Timing:PE + Valence:PE + Timing:Valence:PE + PHQ:PE + Timing:PHQ:PE + Valence:PHQ:PE + Timing:Valence:PHQ:PE + Electrode + Timing:Electrode + Valence:Electrode + Timing:Valence:Electrode + PHQ:Electrode + Timing:PHQ:Electrode + Valence:PHQ:Electrode + Timing:Valence:PHQ:Electrode + PE:Electrode + Timing:PE:Electrode + Valence:PE:Electrode + Timing:Valence:PE:Electrode + PHQ:PE:Electrode + Timing:PHQ:PE:Electrode + Valence:PHQ:PE:Electrode + Timing:Valence:PHQ:PE:Electrode + (1 + Electrode + Timing + Valence + Valence:Electrode + Timing:Electrode + PE \| Participant)* |
|  |  |
| Familial vulnerability | *N170 amplitude ~ 1 + Timing + Valence + Timing:Valence + Vulnerability + Timing:Vulnerability + Valence:Vulnerability + Timing:Valence:Vulnerability + PE + Timing:PE + Valence:PE + Timing:Valence:PE + Vulnerability:PE + Timing:Vulnerability:PE + Valence:Vulnerability:PE + Timing:Valence:Vulnerability:PE + Electrode + Timing:Electrode + Valence:Electrode + Timing:Valence:Electrode + Vulnerability:Electrode + Timing:Vulnerability:Electrode + Valence:Vulnerability:Electrode + Timing:Valence:Vulnerability:Electrode + PE:Electrode + Timing:PE:Electrode + Valence:PE:Electrode + Timing:Valence:PE:Electrode + Vulnerability:PE:Electrode + Timing:Vulnerability:PE:Electrode + Valence:Vulnerability:PE:Electrode + Timing:Valence:Vulnerability:PE:Electrode + (1 + Electrode + Timing + Valence + Valence:Electrode + Timing:Electrode + PE + Valence:PE \| Participant)* |
| *Note.* LME = linear mixed effects. BDI = BDI-II (mean centered, yielding negative values for scores below the mean vs. positive values for scores above the mean), PHQ = modified PHQ-9 (mean centered, yielding negative values for scores below the mean vs. positive values for scores above the mean), Vulnerability = Familial Vulnerability (first-degree relatives without a history of depression [−0.5] vs. first-degree relatives with a depression diagnosis [0.5]), Timing = Feedback Timing (immediate [-0.5] vs. delayed [0.5]), Valence = Feedback Valence (negative [-0.5] vs. positive [0.5]), PE = unsigned PE (scaled and mean centered, yielding negative values for PE values below the mean vs. positive values for PE values above the mean), Electrode (P7 [-0.5] vs. P8 [0.5]). | |

| **Table S5** | | | | | | |
| --- | --- | --- | --- | --- | --- | --- |
| *Results for the GLME analyses on accuracy* | | | | | | |
| Model | Effect | β-estimate | *SE* | *z* | *p* |  |
| Current depressive symptoms (BDI) | Block | 1.42 | 0.23 | 6.04 | <.001 | *** |
|  | BDI | -0.04 | 0.02 | -2.55 | .011 | * |
|  | Timing | 0.11 | 0.13 | 0.81 | .419 |  |
|  | Block:BDI | -0.04 | 0.02 | -1.62 | .106 |  |
|  | BDI-II:Timing | -0.02 | 0.01 | -1.41 | .160 |  |
|  | Block:Timing | -0.34 | 0.32 | -1.08 | .280 |  |
|  | Block:BDI:Timing | 0.01 | 0.03 | 0.16 | .870 |  |
| Past depressive episodes (PHQ) | Block | 1.40 | 0.24 | 5.97 | <.001 | *** |
|  | PHQ | -0.05 | 0.03 | -1.79 | .073 |  |
|  | Timing | 0.10 | 0.13 | 0.72 | .474 |  |
|  | Block:PHQ | -0.02 | 0.04 | -0.54 | .593 |  |
|  | Block:Timing | -0.37 | 0.32 | -1.18 | .239 |  |
|  | PHQ:Timing | 0.00 | 0.02 | -0.01 | .989 |  |
|  | Block:PHQ:Timing | 0.09 | 0.05 | 1.65 | .099 |  |
| Familial vulnerability | Block | 1.24 | 0.33 | 3.76 | <.001 | *** |
|  | Vulnerability | -0.48 | 0.45 | -1.07 | .285 |  |
|  | Timing | -0.09 | 0.16 | -0.56 | .573 |  |
|  | Block:Timing | -0.46 | 0.43 | -1.05 | .294 |  |
|  | Vulnerability:Timing | -0.23 | 0.31 | -0.73 | .467 |  |
|  | Block:Vulnerability | -0.55 | 0.65 | -0.84 | .399 |  |
|  | Block:Vulnerability:Timing | 0.48 | 0.85 | 0.57 | .569 |  |
| *Note.* *n* = 45. GLME = generalized linear mixed effects, *SE* = standard error, Timing = Feedback Timing, BDI = BDI-II, PHQ = modified PHQ-9, Vulnerability = Familial Vulnerability. The sign of the β-estimates indicates the direction of main effects for the fixed-effects predictors BDI (mean centered, yielding negative values for scores below the mean vs. positive values for scores above the mean), PHQ (mean centered, yielding negative values for scores below the mean vs. positive values for scores above the mean), Familial Vulnerability (first-degree relatives without a history of depression [−0.5] vs. first-degree relatives with a depression diagnosis [0.5]), Block (1 [-0.5], 2 [-0.167], 3 [0.167], 4 [0.5]), and Feedback Timing (immediate [-0.5] vs. delayed [0.5]).  ****p* < .001, **p* < .050 | | | | | | |

| **Table S6** |  |  |  |  |  |  |
| --- | --- | --- | --- | --- | --- | --- |
| *Results for the LME analysis on the FRN amplitude including BDI* | | | | | | |
| Effect | β-estimate | *SE* | *df* | *t* | *p* |  |
| Timing | 0.87 | 0.29 | 54.59 | 3.02 | .004 | ** |
| Valence | 2.22 | 0.23 | 41.42 | 9.74 | <.001 | *** |
| BDI | -0.07 | 0.05 | 42.15 | -1.29 | .205 |  |
| PE | 0.89 | 0.31 | 25.44 | 2.90 | .008 | ** |
| Timing:Valence | -0.74 | 0.28 | 13417.45 | -2.65 | .008 | ** |
| Timing:BDI | -0.06 | 0.03 | 50.64 | -1.89 | .065 |  |
| Valence:BDI | 0.00 | 0.02 | 38.37 | 0.10 | .920 |  |
| Timing:PE | -0.28 | 0.45 | 13147.85 | -0.63 | .528 |  |
| Valence:PE | 5.73 | 0.88 | 26.47 | 6.54 | <.001 | *** |
| BDI:PE | 0.02 | 0.03 | 27.25 | 0.47 | .643 |  |
| Timing:Valence:BDI | -0.01 | 0.03 | 13808.02 | -0.32 | .749 |  |
| Timing:Valence:PE | 0.15 | 0.99 | 7036.57 | 0.15 | .882 |  |
| Timing:BDI:PE | 0.01 | 0.05 | 12738.57 | 0.16 | .871 |  |
| Valence:BDI:PE | -0.10 | 0.10 | 26.87 | -1.07 | .293 |  |
| Timing:Valence:BDI:PE | -0.12 | 0.11 | 8801.38 | -1.04 | .300 |  |
| *Note.* *n* = 45. LME = linear mixed effects, *SE* = standard error, *df* = degrees of freedom, Timing = Feedback Timing, Valence = Feedback Valence, BDI = BDI-II, PE = unsigned PE. The sign of the β-estimates indicates the direction of main effects for the fixed-effects predictors Feedback Timing (immediate [-0.5] vs. delayed [0.5]), Feedback Valence (negative [-0.5] vs. positive [0.5]), BDI (mean centered, yielding negative values for scores below the mean vs. positive values for scores above the mean) and PE (scaled and mean centered, yielding negative values for PE values below the mean vs. positive values for PE values above the mean).  *** *p* < .001, ** *p* < .010 | | | | | | |

| **Table S7** |  |  |  |  |  |  |
| --- | --- | --- | --- | --- | --- | --- |
| *Results for the LME analysis on the FRN amplitude including PHQ* | | | | |  |  |
|  | β-estimate | *SE* | *df* | *t* | *P* |  |
| Timing | 0.85 | 0.29 | 53.93 | 2.91 | .005 | ** |
| Valence | 2.22 | 0.23 | 40.65 | 9.83 | <.001 | *** |
| PHQ | -0.08 | 0.09 | 42.67 | -0.90 | .374 |  |
| PE | 0.89 | 0.31 | 25.17 | 2.88 | .008 | ** |
| Timing:Valence | -0.73 | 0.28 | 13469.55 | -2.63 | .009 | ** |
| Timing:PHQ | -0.06 | 0.05 | 54.60 | -1.11 | .271 |  |
| Valence:PHQ | -0.02 | 0.04 | 42.34 | -0.58 | .562 |  |
| Timing:PE | -0.30 | 0.45 | 13144.80 | -0.67 | .504 |  |
| Valence:PE | 5.73 | 0.89 | 28.96 | 6.42 | <.001 | *** |
| PHQ:PE | -0.01 | 0.05 | 21.86 | -0.10 | .924 |  |
| Timing:Valence:PHQ | -0.03 | 0.05 | 13466.57 | -0.67 | .503 |  |
| Timing:Valence:PE | 0.04 | 0.99 | 7177.33 | 0.04 | .971 |  |
| Timing:PHQ:PE | -0.03 | 0.07 | 13597.04 | -0.34 | .737 |  |
| Valence:PHQ:PE | -0.12 | 0.15 | 26.12 | -0.81 | .425 |  |
| Timing:Valence:PHQ:PE | -0.03 | 0.16 | 9540.55 | -0.16 | .873 |  |
| *Note. n* = 45. LME = linear mixed effects, *SE* = standard error, *df* = degrees of freedom, Timing = Feedback Timing, Valence = Feedback Valence, PHQ = modified PHQ-9, PE = unsigned PE. The sign of the β-estimates indicates the direction of main effects for the fixed-effects predictors Feedback Timing (immediate [-0.5] vs. delayed [0.5]), Feedback Valence (negative [-0.5] vs. positive [0.5]), PHQ (mean centered, yielding negative values for scores below the mean vs. positive values for scores above the mean), and PE (scaled and mean centered, yielding negative values for PE values below the mean vs. positive values for PE values above the mean).  *** *p* < .001, ** *p* < .010, * *p* < .050 | | | | | | |

**FRN Analysis Including PHQ**

The LME analysis on the FRN amplitude including PHQ as a predictor alongside Feedback Timing, Feedback Valence and PE replicated all of the effects described for the model containing the BDI in the main text. The analysis replicated the significant effect of Feedback Valence (*p* < .001), with more negative amplitudes following negative compared to positive feedback. In addition, the significant effect of Feedback Timing (*p* = .005), indicating more negative amplitudes following immediate compared to delayed feedback, was replicated. Again, a significant interaction between Feedback Valence and Feedback Timing (*p* = .009) explained these effects further: Negative feedback was associated with more negative amplitudes for both immediate (β = 2.59, *SE* = 0.26, *t* = 9.90, *p* < .001) and delayed feedback (β = 1.86, *SE* = 0.27, *t* = 6.88, *p* < .001), but the effect was stronger for immediate feedback. Furthermore, we could replicate a significant effect of PE (*p* = .008) that was again further explained by a significant interaction between PE and Feedback Valence (*p* < .001), which we thus resolved. There was a significant effect of PE on the FRN amplitude for negative feedback, with more negative amplitudes for more unexpected feedback (β = -1.98, *SE* = 0.51, *t* = -3.91, *p* = .002). For positive feedback, this effect was reversed with more positive amplitudes for more unexpected feedback (β = 3.75, *SE* = 0.58, *t* = 6.51, *p* < .001). All other effects (including effects involving the PHQ) were not significant (all *p*s ≥ .271; see Table S11 above for β-estimates and effect-specific *t*-tests).

| **Table S8** |  |  |  |  |  |  |
| --- | --- | --- | --- | --- | --- | --- |
| *Results for the LME analysis on the FRN amplitude including Familial Vulnerability* | | | | | |  |
| Effect | β-estimate | *SE* | *df* | *t* | *p* |  |
| Timing | 1.19 | 0.41 | 40.75 | 2.87 | .007 | ** |
| Valence | 2.36 | 0.29 | 29.38 | 8.24 | <.001 | *** |
| Vulnerability | 2.47 | 1.25 | 35.59 | 1.97 | .056 |  |
| PE | 0.80 | 0.38 | 15.57 | 2.13 | .050 | * |
| Timing:Valence | -1.00 | 0.36 | 11014.83 | -2.77 | .006 | ** |
| Timing:Vulnerability | 1.31 | 0.83 | 40.75 | 1.58 | .122 |  |
| Valence:Vulnerability | -0.26 | 0.57 | 29.38 | -0.46 | .647 |  |
| Timing:PE | -0.37 | 0.57 | 11135.99 | -0.65 | .518 |  |
| Valence:PE | 4.60 | 0.64 | 11259.45 | 7.22 | <.001 | *** |
| Vulnerability:PE | -1.27 | 0.76 | 15.57 | -1.69 | .111 |  |
| Timing:Valence:Vulnerability | -0.92 | 0.72 | 11014.83 | -1.28 | .202 |  |
| Timing:Valence:PE | 0.58 | 1.26 | 8485.11 | 0.46 | .645 |  |
| Timing:Vulnerability:PE | 0.20 | 1.15 | 11135.99 | 0.18 | .859 |  |
| Valence:Vulnerability:PE | 0.84 | 1.27 | 11259.45 | 0.66 | .509 |  |
| Timing:Valence:Vulnerability:PE | 1.72 | 2.51 | 8485.11 | 0.69 | .492 |  |
| *Note. n* = 37. LME = linear mixed effects, *SE* = standard error, *df* = degrees of freedom, Timing = Feedback Timing, Valence = Feedback Valence, Vulnerability = Familial Vulnerability, PE = unsigned PE. The sign of the β-estimates indicates the direction of main effects for the fixed-effects predictors Feedback Timing (immediate [-0.5] vs. delayed [0.5]), Feedback Valence (negative [-0.5] vs. positive [0.5]), Vulnerability (first-degree relatives without a history of depression [−0.5] vs. first-degree relatives with a depression diagnosis [0.5]), and PE (scaled and mean centered, yielding negative values for PE values below the mean vs. positive values for PE values above the mean).  *** *p* < .001, * *p* < .050 | | | | | | |

**FRN Analysis Including Familial Vulnerability**

The LME analysis on the FRN amplitude including Familial Vulnerability as a predictor alongside Feedback Timing, Feedback Valence and PE replicated all of the effects described for the model containing the BDI in the main text. The analysis replicated the significant effect of Feedback Valence (*p* < .001), with more negative amplitudes following negative compared to positive feedback. In addition, the significant effect of Feedback Timing (*p* = .007), indicating more negative amplitudes following immediate compared to delayed feedback, was replicated. Again, a significant interaction between Feedback Valence and Feedback Timing (*p* = .006) explained these effects further: Negative feedback was associated with more negative amplitudes for both immediate (β = 2.82, *SE* = 0.28, *t* = 10.03, *p* < .001) and delayed feedback (β = 2.08, *SE* = 0.29, *t* = 7.23, *p* < .001), but the effect was stronger for immediate feedback. The effect of PE was at the threshold of significance (*p* = .050), but we were able to replicate the significant interaction between PE and Feedback Valence (*p* < .001), which we thus resolved. There was a significant effect of PE on the FRN amplitude for negative feedback, with more negative amplitudes for more unexpected feedback (β = -1.01, *SE* = 0.43, *t* = -2.37, *p* = .044). For positive feedback, this effect was reversed with more positive amplitudes for more unexpected feedback (β = 3.35, *SE* = 0.43, *t* = 7.72, *p* < .001). All other effects (including effects involving Familial Vulnerability) were not significant (all *p*s ≥ .056; see Table S12 above for β-estimates and effect-specific *t*-tests).

| **Table S9** | | | | | | |
| --- | --- | --- | --- | --- | --- | --- |
| *Results for the LME analysis on the N170 amplitude including BDI* | | | | | | |
| Effect | β-estimate | *SE* | *df* | *t* | *p* |  |
| Timing | 0.47 | 0.36 | 49.05 | 1.31 | .198 |  |
| Valence | 0.47 | 0.25 | 38.14 | 1.91 | .063 |  |
| BDI | 0.03 | 0.06 | 43.34 | 0.46 | .649 |  |
| PE | -0.45 | 0.26 | 15.42 | -1.74 | .102 |  |
| Electrode | -2.49 | 0.90 | 43.95 | -2.75 | .009 | ** |
| Timing:Valence | 0.28 | 0.26 | 23157.78 | 1.06 | 0.291 |  |
| Timing:BDI | 0.03 | 0.04 | 46.99 | 0.65 | .518 |  |
| Valence:BDI | 0.01 | 0.03 | 35.96 | 0.48 | .632 |  |
| Timing:PE | 0.09 | 0.42 | 24156.31 | 0.21 | .832 |  |
| Valence:PE | -2.80 | 0.48 | 22604.02 | -5.85 | <.001 | *** |
| BDI:PE | 0.06 | 0.03 | 16.94 | 1.89 | .077 |  |
| Timing:Electrode | -0.02 | 0.40 | 66.47 | -0.06 | .954 |  |
| Valence:Electrode | 2.16 | 0.40 | 64.66 | 5.46 | <.001 | *** |
| BDI:Electrode | -0.09 | 0.10 | 43.64 | -0.92 | .363 |  |
| PE:Electrode | -0.26 | 0.45 | 3464.48 | -0.58 | .565 |  |
| Timing:Valence:BDI | 0.02 | 0.03 | 26194.06 | 0.83 | .404 |  |
| Timing:Valence:PE | -0.15 | 0.94 | 17003.06 | -0.16 | .870 |  |
| Timing:BDI:PE | 0.10 | 0.05 | 22050.20 | 1.98 | .047 | * |
| Valence:BDI:PE | 0.09 | 0.05 | 24532.31 | 1.68 | .093 |  |
| Timing:Valence:Electrode | 0.03 | 0.52 | 27505.85 | 0.05 | .957 |  |
| Timing:BDI:Electrode | 0.00 | 0.04 | 59.16 | -0.02 | .981 |  |
| Valence:BDI:Electrode | 0.04 | 0.04 | 57.95 | 0.90 | .370 |  |
| Timing:PE:Electrode | 0.12 | 0.84 | 26241.62 | 0.15 | .884 |  |
| Valence:PE:Electrode | 1.10 | 0.95 | 25900.45 | 1.16 | .246 |  |
| BDI:PE:Electrode | 0.05 | 0.05 | 4822.82 | 0.91 | .364 |  |
| Timing:Valence:BDI:PE | -0.07 | 0.11 | 20110.07 | -0.66 | .507 |  |
| Timing:Valence:BDI:Electrode | -0.03 | 0.05 | 27695.23 | -0.61 | .539 |  |
| Timing:Valence:PE:Electrode | -2.18 | 1.82 | 4225.98 | -1.20 | .232 |  |
| Timing:BDI:PE:Electrode | -0.09 | 0.10 | 26779.66 | -0.94 | .345 |  |
| Valence:BDI:PE:Electrode | 0.03 | 0.11 | 26754.40 | 0.28 | .780 |  |
| Timing:Valence:BDI:PE:Electrode | -0.42 | 0.21 | 5828.78 | -2.05 | .041 | * |
| *Note.* *n* = 45. LME = linear mixed effects, *SE* = standard error, *df* = degrees of freedom, Timing = Feedback Timing, Valence = Feedback Valence, BDI = BDI-II, PE = unsigned PE. The sign of the β-estimates indicates the direction of main effects for the fixed-effects predictors Feedback Timing (immediate [-0.5] vs. delayed [0.5]), Feedback Valence (negative [-0.5] vs. positive [0.5]), BDI (mean centered, yielding negative values for scores below the mean vs. positive values for scores above the mean), PE (scaled and mean centered, yielding negative values for PE values below the mean vs. positive values for PE values above the mean) and Electrode (P7 [-0.5] vs. P8 [0.5]).  *** *p* < .001, ** *p* < .010, * *p* < .050 | | | | | | |

| **Table S10** |  |  |  |  |  |  |
| --- | --- | --- | --- | --- | --- | --- |
| *Simple slope analyses for the interaction between BDI, Feedback Timing and PE found in the LME analysis on the N170* | | | | | | |
| BDI Scores | Feedback Timing | β-estimate | *SE* | *t* | *p* |  |
| Low (= -1 *SD*) | Immediate | -0.82 | 0.50 | -1.64 | .648 |  |
|  | Delayed | -1.58 | 0.48 | -3.30 | .012 | * |
| Medium (= Mean) | Immediate | -0.68 | 0.35 | -1.97 | .330 |  |
|  | Delayed | -0.60 | 0.33 | -1.80 | .474 |  |
| High (= +1 *SD*) | Immediate | -0.54 | 0.50 | -1.07 | >.999 |  |
|  | Delayed | 0.38 | 0.47 | 0.80 | >.999 |  |
| Note. *n* = 45. PE = unsigned PE, BDI = BDI-II, *SD* = standard deviation, *SE* = standard error. Presented *p*-values were Bonferroni-corrected. The sign of the β-estimates indicates the direction of unsigned PE (scaled and mean centered, yielding negative values for PE values below the mean vs. positive values for PE values above the mean) effects on N170 amplitudes in the respective condition.  **p* < .050 | | | | | | |

| **Table S11** | | | | | | | | | |
| --- | --- | --- | --- | --- | --- | --- | --- | --- | --- |
| *Simple slope analyses for the interaction between Electrode, BDI, Feedback Timing, Feedback Valence and PE found in the LME analysis on the N170* | | | | | | | | | |
| Electrode | BDI | Timing | Valence | β-estimate | *SE* | *df* | *t* | *p* |  |
| P7 | High | Delayed | Positive | -0.15 | 0.91 | 641.56 | -0.16 | >.999 |  |
|  |  |  | Negative | 1.41 | 0.89 | 553.91 | 1.58 | >.999 |  |
|  |  | Immediate | Positive | -2.58 | 0.98 | 745.34 | -2.64 | .134 |  |
|  |  |  | Negative | 1.19 | 0.93 | 613.52 | 1.28 | >.999 |  |
|  | Low | Delayed | Positive | -3.36 | 0.92 | 437.12 | -3.67 | .004 | ** |
|  |  |  | Negative | 0.86 | 0.94 | 626.81 | 0.91 | >.999 |  |
|  |  | Immediate | Positive | -1.92 | 0.99 | 688.34 | -1.94 | .853 |  |
|  |  |  | Negative | 1.95 | 0.95 | 563.98 | 2.05 | .650 |  |
| P8 | High | Delayed | Positive | -1.16 | 0.91 | 612.82 | -1.28 | >.999 |  |
|  |  |  | Negative | 2.04 | 0.90 | 530.50 | 2.26 | .384 |  |
|  |  | Immediate | Positive | 0.14 | 0.98 | 772.55 | 0.14 | >.999 |  |
|  |  |  | Negative | -0.48 | 0.94 | 575.24 | -0.52 | >.999 |  |
|  | Low | Delayed | Positive | -2.73 | 0.92 | 418.39 | -2.97 | .051 |  |
|  |  |  | Negative | -0.18 | 0.95 | 618.58 | -0.19 | >.999 |  |
|  |  | Immediate | Positive | -3.09 | 1.00 | 736.17 | -3.09 | .034 | * |
|  |  |  | Negative | 0.80 | 0.96 | 508.64 | 0.83 | >.999 |  |
| *Note.* *n* = 45. PE = unsigned PE, BDI = BDI-II, Timing = Feedback Timing, Valence = Feedback Valence, *SE* = standard error, *df* = degrees of freedom. Presented *p*-values were Bonferroni-corrected. The sign of the β-estimates indicates the direction of unsigned PE (scaled and mean centered, yielding negative values for PE values below the mean vs. positive values for PE values above the mean) effects on N170 amplitudes in the respective condition.  ***p* < .010, **p* < .050 | | | | | | | | | |

| **Table S12** | | | | | | |
| --- | --- | --- | --- | --- | --- | --- |
| *Results for the LME analysis on the N170 amplitude including PHQ* | | | | | | |
| Effect | β-estimate | *SE* | *df* | *t* | *p* |  |
| Timing | 0.48 | 0.37 | 48.73 | 1.31 | .197 |  |
| Valence | 0.53 | 0.24 | 39.25 | 2.19 | .034 | * |
| PHQ | 0.07 | 0.11 | 43.52 | 0.66 | .516 |  |
| PE | -0.27 | 0.28 | 22.56 | -0.96 | .348 |  |
| Electrode | -2.50 | 0.92 | 43.88 | -2.74 | .009 | ** |
| Timing:Valence | 0.33 | 0.26 | 24621.51 | 1.26 | .207 |  |
| Timing:PHQ | 0.01 | 0.07 | 49.02 | 0.14 | .887 |  |
| Valence:PHQ | -0.06 | 0.04 | 40.42 | -1.44 | .157 |  |
| Timing:PE | 0.06 | 0.42 | 24861.91 | 0.14 | .891 |  |
| Valence:PE | -2.73 | 0.48 | 25107.91 | -5.70 | <.001 | *** |
| PHQ:PE | 0.02 | 0.05 | 19.29 | 0.50 | .623 |  |
| Timing:Electrode | -0.07 | 0.40 | 65.51 | -0.18 | .861 |  |
| Valence:Electrode | 2.18 | 0.39 | 64.67 | 5.56 | <.001 | *** |
| PHQ:Electrode | 0.06 | 0.16 | 43.91 | 0.35 | .730 |  |
| PE:Electrode | -0.23 | 0.45 | 3181.98 | -0.51 | .611 |  |
| Timing:Valence:PHQ | -0.02 | 0.05 | 24719.79 | -0.37 | .714 |  |
| Timing:Valence:PE | -0.09 | 0.95 | 19006.54 | -0.09 | .926 |  |
| Timing:PHQ:PE | -0.01 | 0.07 | 26354.21 | -0.07 | .942 |  |
| Valence:PHQ:PE | 0.15 | 0.08 | 22986.82 | 1.93 | .054 |  |
| Timing:Valence:Electrode | 0.00 | 0.52 | 27523.97 | 0.01 | .993 |  |
| Timing:PHQ:Electrode | 0.02 | 0.07 | 67.95 | 0.25 | .801 |  |
| Valence:PHQ:Electrode | 0.09 | 0.07 | 67.25 | 1.25 | .216 |  |
| Timing:PE:Electrode | 0.18 | 0.84 | 26131.73 | 0.22 | .830 |  |
| Valence:PE:Electrode | 1.07 | 0.95 | 25862.22 | 1.13 | .258 |  |
| PHQ:PE:Electrode | 0.07 | 0.07 | 5801.38 | 0.90 | .368 |  |
| Timing:Valence:PHQ:PE | 0.03 | 0.15 | 22968.14 | 0.21 | .834 |  |
| Timing:Valence:PHQ:Electrode | -0.01 | 0.09 | 27680.67 | -0.08 | .934 |  |
| Timing:Valence:PE:Electrode | -2.16 | 1.82 | 4002.55 | -1.19 | .234 |  |
| Timing:PHQ:PE:Electrode | 0.01 | 0.14 | 27049.30 | 0.05 | .957 |  |
| Valence:PHQ:PE:Electrode | 0.24 | 0.15 | 27105.95 | 1.59 | .113 |  |
| Timing:Valence:PHQ:PE:Electrode | -0.13 | 0.30 | 7134.45 | -0.43 | .670 |  |
| *Note. n* = 45. LME = linear mixed effects, *SE* = standard error, *df* = degrees of freedom, Timing = Feedback Timing, Valence = Feedback Valence, PHQ = modified PHQ-9, PE = unsigned PE. The sign of the β-estimates indicates the direction of main effects for the fixed-effects predictors Feedback Timing (immediate [-0.5] vs. delayed [0.5]), Feedback Valence (negative [-0.5] vs. positive [0.5]), PHQ (mean centered, yielding negative values for scores below the mean vs. positive values for scores above the mean), PE (scaled and mean centered, yielding negative values for PE values below the mean vs. positive values for PE values above the mean) and Electrode (P7 [-0.5] vs. P8 [0.5]).  *** *p* < .001, ** *p* < .010, * *p* < .050 | | | | | | |
|  | | | | | | |

**N170 Analysis Including PHQ**

The LME analysis on the N170 amplitude including PHQ as a predictor alongside Feedback Timing, Feedback Valence, PE and Electrode replicated all of the effects described for the model containing the BDI in the main text. Again, there was a significant effect of electrode (*p* = .009) with more pronounced amplitudes over P8, as well as a significant two-way interaction between Feedback Valence and Electrode (*p* < .001). As for the BDI model, we resolved the interaction via simple slope analyses and found no significant effect of Feedback Valence for P7 (β = -0.56, *SE* = 0.29, *t* = -1.93, *p* = .118), but for P8 negative feedback led to significantly larger N170 amplitudes than positive feedback (β = 1.62, *SE* = 0.33, *t* = 4.90, *p* < .001). In addition, the analysis also replicated the significant interaction between PE and Feedback Valence (*p* < .001), with amplitudes increasing for more unexpected positive feedback (β = -1.64, *SE* = 0.38, *t* = -4.36, *p* < .001) and decreasing for more unexpected negative feedback (β = 1.09, *SE* = 0.37, *t* = 2.97, *p* = .008). In addition, the model revealed a significant effect of Feedback Valence (*p* = .034), with more pronounced amplitudes for negative compared to positive feedback. All other effects were not significant (all *p*s ≥ .054, see Table S8 above for β-estimates and effect-specific *t*-tests).

| **Table S13** |  |  |  |  |  |  |
| --- | --- | --- | --- | --- | --- | --- |
| *Results for the LME analysis on the N170 amplitude including Familial Vulnerability* | | | | | |  |
| Effect | β-estimate | *SE* | *df* | *t* | *p* |  |
| Timing | 0.43 | 0.53 | 37.68 | 0.82 | .415 |  |
| Valence | 0.10 | 0.31 | 30.62 | 0.34 | .739 |  |
| Vulnerability | 1.61 | 1.68 | 34.89 | 0.96 | .343 |  |
| PE | -0.82 | 0.40 | 20.55 | -2.08 | .050 |  |
| Electrode | -2.64 | 1.31 | 35.45 | -2.02 | .051 |  |
| Timing:Valence | 0.18 | 0.34 | 21408.96 | 0.54 | .589 |  |
| Timing:Vulnerability | -0.45 | 1.05 | 37.68 | -0.43 | .669 |  |
| Valence:Vulnerability | -1.67 | 0.62 | 30.62 | -2.71 | .011 | * |
| Timing:PE | -0.14 | 0.53 | 21449.25 | -0.26 | .799 |  |
| Valence:PE | -2.69 | 1.00 | 17.62 | -2.70 | .015 | * |
| Vulnerability:PE | -1.56 | 0.79 | 20.55 | -1.96 | .064 |  |
| Timing:Electrode | 0.68 | 0.49 | 49.16 | 1.38 | .173 |  |
| Valence:Electrode | 2.52 | 0.52 | 47.41 | 4.84 | <.001 | *** |
| Vulnerability:Electrode | -0.11 | 2.62 | 35.45 | -0.04 | .967 |  |
| PE:Electrode | -0.04 | 0.56 | 4260.73 | -0.08 | .938 |  |
| Timing:Valence:Vulnerability | 0.38 | 0.67 | 21408.96 | 0.57 | .572 |  |
| Timing:Valence:PE | -0.24 | 1.18 | 16148.09 | -0.20 | .840 |  |
| Timing:Vulnerability:PE | -0.39 | 1.07 | 21449.25 | -0.36 | .716 |  |
| Valence:Vulnerability:PE | 1.17 | 2.00 | 17.62 | 0.59 | .564 |  |
| Timing:Valence:Electrode | -0.04 | 0.67 | 22686.77 | -0.06 | .954 |  |
| Timing:Vulnerability:Electrode | 1.82 | 0.99 | 49.16 | 1.84 | .072 |  |
| Valence:Vulnerability:Electrode | -0.39 | 1.04 | 47.41 | -0.37 | .711 |  |
| Timing:PE:Electrode | 0.62 | 1.06 | 21846.76 | 0.58 | .560 |  |
| Valence:PE:Electrode | 0.99 | 1.18 | 21180.96 | 0.84 | .400 |  |
| Vulnerability:PE:Electrode | -0.12 | 1.13 | 4260.73 | -0.11 | .912 |  |
| Timing:Valence:Vulnerability:PE | -1.60 | 2.36 | 16148.10 | -0.68 | .498 |  |
| Timing:Valence:Vulnerability:Electrode | 0.67 | 1.34 | 22686.77 | 0.50 | .616 |  |
| Timing:Valence:PE:Electrode | -1.55 | 2.25 | 3450.50 | -0.69 | .492 |  |
| Timing:Vulnerability:PE:Electrode | 2.54 | 2.13 | 21846.76 | 1.20 | .232 |  |
| Valence:Vulnerability:PE:Electrode | 0.92 | 2.36 | 21180.96 | 0.39 | .696 |  |
| Timing:Valence:Vulnerability:PE:Electrode | 1.64 | 4.50 | 3450.50 | 0.36 | .716 |  |
| *Note. n* = 37. LME = linear mixed effects, *SE* = standard error, *df* = degrees of freedom, Timing = Feedback Timing, Valence = Feedback Valence, Vulnerability = Familial Vulnerability, PE = unsigned PE. The sign of the β-estimates indicates the direction of main effects for the fixed-effects predictors Feedback Timing (immediate [-0.5] vs. delayed [0.5]), Feedback Valence (negative [-0.5] vs. positive [0.5]), Vulnerability (first-degree relatives without a history of depression [−0.5] vs. first-degree relatives with a depression diagnosis [0.5]), PE (scaled and mean centered, yielding negative values for PE values below the mean vs. positive values for PE values above the mean) and Electrode (P7 [-0.5] vs. P8 [0.5]).  *** *p* < .001, * *p* < .050 | | | | | | |

**N170 Analysis Including Familial Vulnerability**

The LME analysis on the N170 amplitude including Familial Vulnerability as a predictor alongside Feedback Timing, Feedback Valence, PE and Electrode replicated the significant interaction between Feedback Valence and Electrode (*p* < .001) that was also described for the analysis involving the BDI reported in the main text. Simple slope analyses revealed significantly larger amplitudes following negative compared to positive feedback for P8 (β = 1.88, *SE* = 0.34, *t* = 5.54, *p* < .001), but not for P7 (β = -0.75, *SE* = 0.33, *t* = -2.26, *p* = .060). The analysis also replicated the significant interaction between PE and Feedback Valence (*p* = .015), however, this time amplitudes significantly increased for more unexpected positive feedback (β = -1.90, *SE* = 0.56, *t* = -3.36, *p* = .004), but did not significantly decrease for more unexpected negative feedback (β = 1.13, *SE* = 0.52, *t* = 2.18, *p* = .086). All other effects (apart from the interaction between Feedback Valence and Familial Vulnerability reported in the main text) did not reach significance (all ps ≥ .050; see Table S9 above for β-estimates and effect-specific *t*-tests).
